# Trichodermanins C–E, New Diterpenes with a Fused 6-5-6-6 Ring System Produced by a Marine Sponge-Derived Fungus

**DOI:** 10.3390/md15060169

**Published:** 2017-06-09

**Authors:** Takeshi Yamada, Mayo Suzue, Takanobu Arai, Takashi Kikuchi, Reiko Tanaka

**Affiliations:** Faculty of Pharmaceutical Sciences, Osaka University of Pharmaceutical Sciences, 4-20-1 Nasahara, Takatsuki, Osaka 569-1142, Japan; e11604@gap.oups.ac.jp (M.S.); e12520@gap.oups.ac.jp (T.A.); t.kikuchi@gly.oups.ac.jp (T.K.); tanakar@gly.oups.ac.jp (R.T.)

**Keywords:** trichodermanins, *Trichoderma harzianum*, marine microorganism, *Halichondria okadai*, diterpenes, 6-5-6-6 ring system, cytotoxicity

## Abstract

Trichodermanins C–E (**1**–**3**), new diterpenes with a rare fused 6-5-6-6 ring system, have been isolated from a fungus *Trichoderma harzianum* OUPS-111D-4 separated from a piece of a marine sponge *Halichondria okadai*, and these chemical structures have been established by spectroscopic analyses using IR, MASS, HRFABMS, and NMR spectra. We established their absolute stereostructures by application of the modified Mosher’s method. In addition, **1** inhibited the growth of cancer cell lines potently.

## 1. Introduction

A number of marine-derived compounds have unique structures, some of which exhibit significant biological activities [1,2]. Our purpose is to seed research into antitumor chemotherapy agent from marine microorganisms, and we have reported many cytotoxic metabolites to date [3,4,5,6]. In this study, we examined the metabolites of a fungus *T. harzianum* separated from a piece of a marine sponge *H. okadai.* We have already reported the isolation, structure determination, and cytotoxicity of tandyukisins A–F [7,8,9]. In this continuing search for cytotoxic metabolites from this fungal strain, we isolated three new compounds, trichodermanins C–E (**1**–**3**), classified as diterpene with a rare fused 6-5-6-6 ring system. Previously reported metabolites consisting of this ring system are trichodermanin A [10], and wickerols A and B, which exhibit anti-influenza activity; however, wickerol B possesses the same structure as trichodermanin A [11]. We herein report the first determination of the absolute configurations of **1**–**3** by application of the modified Mosher’s method [12]. In addition, we describe the first examination of the cytotoxic activities of trichodermanins C–E (**1**–**3**).

## 2. Results and Discussion

**Figure 1:**
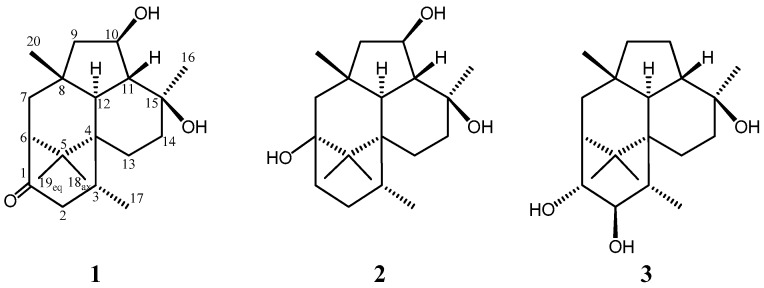
Structures of trichodermanins C–E (**1**–**3**).

This fungus was incubated at 27 °C for 6 weeks in a medium (70 L) containing 1% glucose, 1% malt extract, and 0.05% peptone in artificial seawater adjusted to pH 7.5. After the filtration of culture broth, it was extracted using ethyl acetate, and the concentrated material was purified by column chromatography using silica gel and octa decyl silyl HPLC to afford trichodermanins C (**1**) (2.5 mg), D (**2**) (0.6 mg), and E (**3**) (1.3 mg) as pale yellow oil, respectively (Figure 1).

**Figure 2:**
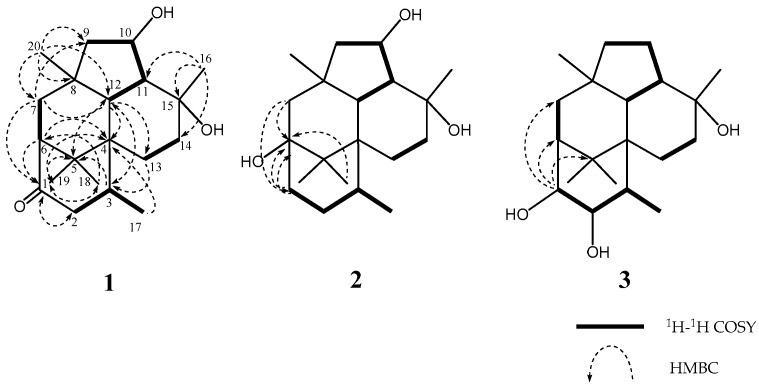
The key ^1^H-^1^H COSY and HMBC correlations of **1**–**3**.

The molecular formula of trichodermanin C (**1**) has been determined as C_20_H_32_O_3_ from the molecular weight 343.2247 [M + Na]^+^ in HRFABMS. Absorptions in the IR spectrum at 3412 and 1694 cm^−1^ indicate the presence of hydroxy and carbonyl groups, respectively. A consideration of the ^1^H and ^13^C NMR spectra of **1** (Table 1 and Table S1) using DEPT and heteronuclear multiple quantum coherence spectroscopy (HMQC) suggested the functional groups as below; i.e., this compound had one secondary methyl (C-17), four tertiary methyls (C-16, C-18, C-19, and C-20), five sp^3^-hybridized methylenes (C-2, C-7, C-9, C-13, and C-14), five sp^3^-methines (C-3, C-6, C-10, C-11, and C-12), one of which is an oxygen-bearing sp^3^-methine (C-10), four quaternary sp^3^-carbons (C-4, C-5, C-8, and C-15), one of which is an oxygen-bearing quaternary sp^3^-carbon (C-15), and one carbonyl group (C-1). ^1^H-^1^H correlation spectroscopy (COSY) revealed four partial structures (Figure 2). In the HMBC spectrum (Figure 2), the correlation from 15-methyl to C-11, C14, and C-15, from 8-methyl to C-7, C-8, C-9, and C-12, from germinal dimethyl (H-18 and H-19) to C-4, C-5, and C-6, and from H-12 to C-4, C-5, and H-13 showed that two cyclohexane rings and a cyclopentane made up a fused 6-5-6 ring system. In addition, the correlation from 3-methyl to C-4, from H-2, H-6, and H-7 to C-1, and from H-12 and H-13 to C-3 revealed the ring junction to C-4 and C-6 of a cyclohexanone ring. This evidence elucidated the planar structure of **1** as shown in Figure 1. The study for the stereochemistry of **1** is described later together with that of **2**.

Trichodermanin D (**2**) was assigned C_20_H_34_O_3_, which contained two more hydrogen atoms than **1**. The NMR spectral features (Table 1 and Table S2) resembled those of **1** except for the proton signal of H-1 (*δ*_H_ 1.90, ddd and *δ*_H_ 1.98, ddd), H-2 (*δ*_H_ 1.64, m and *δ*_H_ 2.12, m), and H-7 (*δ*_H_ 1.56, m and *δ*_H_ 1.62, m), and the carbon signals of C-1 (*δ*_C_ 35.5), C-2 (*δ*_C_ 29.5), C-5 (*δ*_C_ 44.1), C-6 (*δ*_C_ 74.9), C-7 (*δ*_C_ 51.2), C-18 (*δ*_C_ 18.3), and C-19 (*δ*_C_ 19.4) in **2**, and suggested that a carbonyl group at C-1 and an sp^3^-methine at C-6 in **1** disappear, and a methylene and an oxygen-bearing quaternary sp^3^-carbon newly appear in **2**. The ^1^H-^1^H COSY correlation between H-1 and H-2 showed that a carbonyl group at C-1 in **1** was replaced with a methylene in **2** (Figure 2). In the HMBC spectrum, the correlations for the 6-5-6-6 skeleton were observed as those of **1** (Table S2). In addition, the HMBC correlation from H-1, H-7, and germinal dimethyl (H-18 and H-19) to C-6, and from H-7 to C-1 revealed that C-6 was a quaternary sp^3^-carbon bearing a hydroxyl group (Figure 2). The above evidence established the planar structure of **2**.

**Figure 3:**
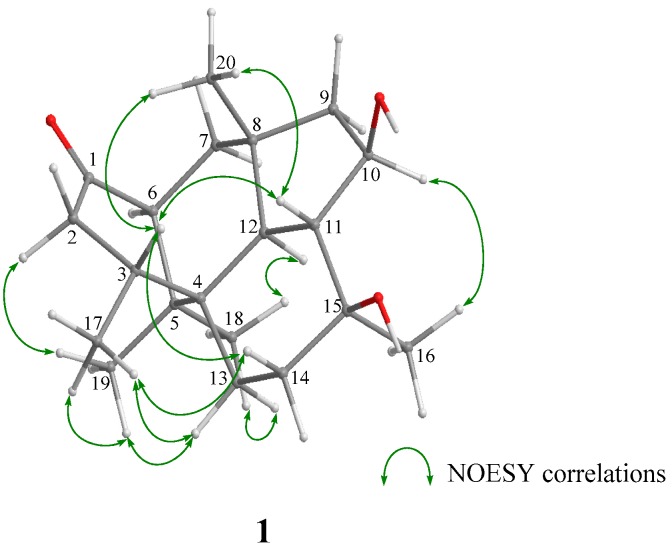
Key NOESY correlations of **1**.

**Figure 4:**
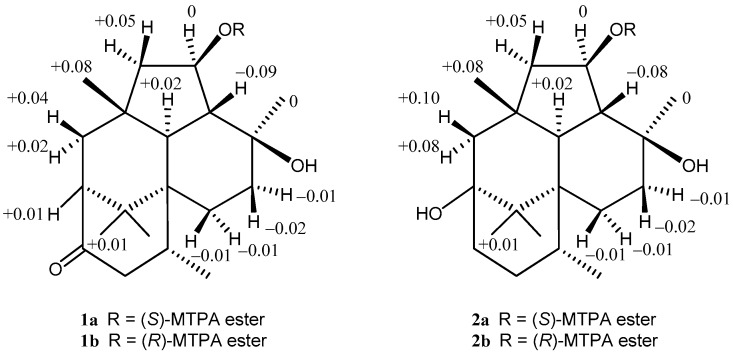
^1^H chemical-shift differences between the (*S*)- and (*R*)-MTPA esters **1a**/**1b**, and **2a**/**2b**, respectively.

For the stereochemistry of **1** and **2**, their relative configurations and conformations were examined by NOESY experiments (Tables S1 and S2, and Figure 3). In the NOESY experiment of **1**, NOESY correlations from H-2α to H-19, and from H-3 to H-11, H-14β, and H-20 showed that the cyclohexenone ring existed in a half boat conformation with 3-CH_3_ in the α-orientation. For the stereochemistry of two cyclohexane rings, NOESY correlations (H-7α/H-12 and H-18, H-11/H-20, H-12/H-16 and H-18, H-13 α/H-18, and H-14β/H-17) demonstrated that 5-CH3 (C-18), H-7α, H-12, 8-CH_3_ (C-20), H-11, H-13a, H-14b, and 15-CH_3_ (C-16) oriented in coaxial arrangements. This revealed that the ring juncture for two cyclohexane rings was *trans*, and both rings existed in a chair conformation, respectively (Figure 3 and Table S1). In addition, the presence of a β-orienting hydroxy group in the cyclopentane was deduced from observed NOESY correlations between H-10 and H-12, and between H-10 and H-16. On the other hand, a detailed examination of NOESY for **2** led to the finding that the relative configuration was the same as that of **1** (Table S2). A significant difference in the structural features of **1** and **2** from wickerols [8] was the presence of a secondary hydroxyl group in the cyclopentane ring; therefore, we applied the modified Mosher’s method [11] to determine their absolute stereostructures. The ^1^H chemical shift differences between the (*S*)- and (*R*)-MTPA esters **1a**/**1b** and **2a**/**2b** revealed an *R* configuration at C-10, respectively (Figure 4).

Trichodermanin E (**3**) had the same molecular formula as **2** by HRFABMS data. In ^1^H and ^13^C NMR spectra of **3**, remarkable differences from those of **2** were observed at some positions (Table 1 and Table S3). The differences in the NMR chemical shifts at H-1 (δH 4.11), H-2 (δH 3.88), H-3 (δH 1.88), H-10 (δH 1.59, 1.80), C-1 (δC 80.4), C-2 (δC 83.7), C-3 (δC 36.6), C-6 (δC 53.2), C-7 δC 40.9), C-9 (δC 43.5), C-10 (δC 21.6), and C-11 (δC 44.2) in **3** from those in **2** were caused by the change in the linkage position of the two hydroxy groups, i.e., **3** was a position isomer of **2**. The ^13^C NMR chemical shifts at C-18 (δC 25.7) and C-19 (δC 25.2) were also different from those of **2**, but were close to those of **1**. The ^1^H-^1^H COSY correlations (H-17/H-3, H-3/H-2, and H-2/H-1) and the chemical shifts of H-1 and H-2 suggested that two hydroxyl groups were present at C-1 and C-2, respectively (Figure 2, Table 1 and Table S3). On the other hand, the correlations (H-9/H-10, H-10/H-11, and H-11/H-12) and the chemical shifts of H-10 showed that the hyroxy methine at C-10 in **1** and **2** replace the methylene in 3 (Figure 2, Table 1 and Table S3). In the HMBC spectrum, the common correlations with those of **1** and **2** led to the construction of the 6-5-6-6 ring system (Table S3). In addition, the HMBC correlation from H-1 to C-5, C-6, and C-7 confirmed the planar structure of **3**. The NOESY correlation between H-1 and H-20 showed that 1-OH oriented to the β-configuration in equatorial arrangement. In addition, the correlations between H-2 and H-19 demonstrated that 2-OH oriented to the β-configuration in equatorial arrangement (Table S3). The analysis of NOESY revealed that the relative configurations of the chiral centers in **3** were identified with those of the above metabolites except for C-1 and C-2; therefore, we deduced the absolute stereostructure of **3**, as shown in Figure 1, together with the consideration that **1**–**3** were metabolites derived from the same fungal strain, as shown in Figure 1.

As a primary screen for antitumor activity, the cancer cell growth inhibitory properties of trichodermanins C–E (**1**–**3**) were examined using murine P388 leukemia, human HL-60 leukemia, and murine L1210 leukemia cell lines. The results were shown in Table 2. Compound **1**, which has a carbonyl group at C-1, exhibited significant cytotoxic activity against these cancer cells. We believe that the discovery of these related metabolites produced by the fungus *T. harzianum* can help us to resolve structure-activity relationships.

## 3. Conclusions

In this study, three new terpenes with a rare fused 6-5-6-6 ring system, trichodermanins C–E (**1**–**3**), were isolated from the fungus *T. harzianum* separated from the marine sponge, *H. okadai*. Spectral analyses and chemical transformation were utilized to elucidate the absolute stereostructures of these compounds. In the cytotoxic assay using three cancer cell lines, **1** exhibited significant activity.

## 4. Experimental Section

### 4.1. General Experimental Procedures

NMR spectra were recorded on an Agilent-NMR-vnmrs600 (Tokyo, Japan) with tetramethylsilane (TMS, Sigma-Aldrich Japan, Tokyo, Japan) as an internal reference. FABMS was recorded using a JEOL JMS-7000 mass spectrometer (Tokyo, Japan). IR spectra was recorded on a JASCO FT/IR-680 Plus (Tokyo, Japan). Optical rotations were measured using a JASCO DIP-1000 digital polarimeter (Tokyo, Japan). Silica gel 60 (230–400 mesh, Nacalai Tesque, Inc., Kyoto, Japan) was used for column chromatography with medium pressure. ODS HPLC was run on a JASCO PU-1586 with a differential refractometer (RI-1531) and Cosmosil Packed Column 5C_18_-MSII (25 cm × 20 mm i.d.). Analytical TLC was performed on precoated Merck aluminium sheets (DC-Alufolien Kieselgel 60 F254, 0.2 mm) with the solvent system CH_2_Cl_2_–MeOH (19:1), and compounds were viewed under a UV lamp and sprayed with 10% H_2_SO_4_ followed by heating.

### 4.2. Fungal Material

In this section, since this study is a follow-up report for this fungal strain, please see the previous reports [7,8,9].

### 4.3. Culturing and Isolation of Metabolites

The EtOAc extract (9.8 g) of the culture filtrate, which was obtained as described in the previous literature [7,8,9], was chromatographed on a silica gel column with a CHCl_3_–MeOH gradient as the eluent to afford Fr.1 (2% MeOH in CHCl_3_ eluate, 493.4 mg) and Fr.2 (5% MeOH in CHCl_3_ eluate, 659.4 mg). Fr.1 was purified by ODS HPLC using MeOH–H_2_O (80:20) as the eluent to afford Fr.3 (11.3 mg). Fr.3 was purified by HPLC using MeCN–H_2_O (40:60) as the eluent to afford **1** (2.5 mg, *t_R_* 28.3 min) and **2** (0.6 mg, *t_R_* 22.0 min). Fr.2 was purified by ODS HPLC using MeOH–H_2_O (80:20) as the eluent to afford Fr.4 (9.8 mg) and **2** (7.2 mg). Fr.4 was purified by HPLC using MeCN–H_2_O (40:60) as the eluent to afford **3** (1.3 mg, *t_R_* 27.6 min).

Trichodermanin C (**1**): pale yellow oil; ${\left[\alpha \right]}_{D}^{22}$ +3.7 (*c* 0.09, MeOH); IR (neat) *ν*_max_/cm^−1^: 3412, 1694. FABMS *m*/*z* (rel. int.): 343 ([M + Na]^+^, 77.1%) 321 ([M + H]^+^, 15.6%), 115 (58.4%). HRFABMS *m*/*z* 343.2247 [M + Na]^+^ (calcd. for C_20_H_32_O_3_Na: 343.2240).

Trichodermanin D (**2**): pale yellow oil; ${\left[\alpha \right]}_{D}^{22}$ +9.9 (*c* 0.04, MeOH); IR (neat) *ν*_max_/cm^−1^: 3284. FABMS *m*/*z* (rel. int.): 345 ([M + Na]^+^, 51.0%) 305 (47.7%), 287 (100%), 147 (74.0%), 115 (90.1%). HRFABMS *m*/*z* 345.2419 [M + Na]^+^ (calcd. for C_20_H_34_O_3_Na: 345.2396).

Trichodermanin E (**3**): pale yellow oil; ${\left[\alpha \right]}_{D}^{22}$ +188.0 (*c* 0.09, MeOH); IR (neat) *ν*_max_/cm^−1^: 3383. FABMS *m*/*z* (rel. int.): 345 ([M + Na]^+^, 37.4%) 305 (43.8%), 287 (54.5%), 115 (100%). HRFABMS *m*/*z* 345.2397 [M + Na]^+^ (calcd. for C_20_H_34_O_3_Na: 345.2396).

### 4.4. Formation of the (S)- and (R)-MTPA Esters of ***1***

To a solution of **1** (2.1 mg, 6.6 µmol) in abs. pyridine (0.3 mL), (*R*)-(−)-MTPA chloride (5.0 mg, 19.8 µmol) was added, and the reaction mixture was stirred at room temperature for 2 h. Water (1.0 mL) was added to the reaction mixture, and extracted using CH_2_Cl_2_. The organic layer was evaporated under reduced pressure, and the residue was purified by HPLC using MeOH–H_2_O (90:10) as the eluent to afford (*S*)-MTPA ester **1a** (1.5 mg, 42.4%) as a colorless oil.

**1** (1.8 mg, 5.6 µmol) and (*S*)-(+)-MTPA chloride (5.0 mg, 19.8 µmol) were treated with the same procedure to afford (*R*)-MTPA ester **1b** (1.3 mg, 36.3%) as a colorless oil.

MTPA ester **1a**: Pale yellow oil; HRFABMS *m/z* 537.2822 [M + H]^+^ (calcd. for C_30_H_40_F_3_O_5_: 537.2848). ^1^H and ^13^C NMR data are listed in Table S4 of Supplementary Materials.

MTPA ester **1b**: Pale yellow oil; HRFABMS *m/z* 537.2827 [M + H]^+^ (calcd. for C_30_H_40_F_3_O_5_: 537.2848). ^1^H and ^13^C NMR data are listed in Table S4 of Supplementary Materials.

### 4.5. Formation of the (S)- and (R)-MTPA Esters of ***2***

The treatment with the same procedure of **2** (both 2.0 mg, 6.3 µmol) yielded (*S*)- and (*R*)-MTPA esters **2a** (1.3 mg, 38.1%) and **2b** (1.4 mg, 41.3%) as colorless oils, respectively.

MTPA ester **2a**: Pale yellow oil; HRFABMS *m/z* 561.2810 [M + Na]^+^ (calcd. for C_30_H_41_F_3_O_5_Na: 561.2801). ^1^H and ^13^C NMR data are listed in Table S5 of Supplementary Materials.

MTPA ester **2b**: Pale yellow oil; HRFABMS *m/z* 561.2810 [M + Na]^+^ (calcd. for C_30_H_41_F_3_O_5_Na: 561.2801). ^1^H and ^13^C NMR data are listed in Table S5 of Supplementary Materials.

### 4.6. Assay for Cytotoxicity

Cytotoxic activities of **1**–**3** were examined with the 3-(4,5-dimethyl-2-thiazolyl)-2,5-diphenyl-2H-tetrazolium bromide (MTT) method. P388, HL-60, and L1210 cells were cultured in Roswell Park Memorial Institute 1640 Medium (10% fetal calf serum) at 37 °C in 5% CO_2_. The test materials were dissolved in dimethyl sulfoxide (DMSO) to give a concentration of 10 mM, and the solution was diluted with the Essential Medium to yield concentrations of 200, 20, and 2 μM, respectively. Each solution was combined with each cell suspension (1 × 10^−5^ cells/mL) in the medium, respectively. After incubating at 37 °C for 72 h in 5% CO_2_, grown cells were labeled with 5 mg/mL MTT in phosphate-buffered saline (PBS), and the absorbance of formazan dissolved in 20% sodium dodecyl sulfate (SDS) in 0.1 N HCl was measured at 540 nm with a microplate reader (MTP-310, CORONA electric, Ibaragi, Japan). Each absorbance values were expressed as a percentage relative to that of the control cell suspension that was prepared without the test substance using the same procedure as that described above. All assays were performed three times, semilogarithmic plots were constructed from the averaged data, and the effective dose of the substance required to inhibit cell growth by 50% (IC_50_) was determined.

## Figures and Tables

**Table 1:** ^1^H and ^13^C NMR spectral data for metabolites (**1**–**3**) in CDCl_3_.

Position	1		2		3	
	*δ*_H_ *^a^*	*δ*_C_	*δ*_H_ *^a^*	*δ*_C_	*δ*_H_ *^a^*	*δ*_C_
1α	-	217.7 (s)	1.90 ddd (14.4, 2.4, 2.4)	35.5 (t)	-	-
1β	-	-	1.98 ddd (14.4, 10.8, 6.0)	-	4.11 d (5.4)	80.4 (d)
2α	2.27 dd (20.4, 7.2)	48.7 (t)	1.64 m	29.5 (t)	3.88 dd (7.8, 5.4)	83.7 (d)
2β	2.91 dd (20.4, 9.0)	-	2.12 m	-	-	-
3	2.44 dqd (9.0,7.2,7.2)	26.1 (d)	2.14 m	26.0 (d)	1.88 qd (7.8, 7.8)	36.6 (d)
4	-	39.5 (s)	-	41.0 (s)	-	41.2 (s)
5	-	38.2 (s)	-	44.1 (s)	-	39.4 (s)
6	2.03 dd (3.6, 3.6)	58.0 (d)	-	74.9 (s)	1.50 dd (4.8, 3.0)	53.2 (d)
7α	1.76 dd (13.8, 3.6)	41.4 (t)	1.56 m	51.2 (t)	1.78 dd (13.8, 4.8)	40.9 (t)
7β	1.92 dd (13.8, 3.6)	-	1.62 m	-	1.70 dd (13.8, 3.0)	-
8	-	39.0 (s)	-	39.1 (s)	-	39.6 (s)
9α	1.50 m	53.9 (t)	1.51 m	54.4 (t)	1.03 m	43.5 (t)
9β	1.50 m	-	1.51 m	-	1.43 m	-
10α	4.41 ddd (7.8, 4.8, 1.2)	72.6 (d)	4.39 ddd (8.4, 4.8, 1.2)	72.8 (d)	1.59 m	21.6 (t)
10β	-	-	-	-	1.80 m	-
11	1.95 dd (12.6, 4.8)	54.7 (d)	1.88 dd (12.6, 4.8)	55.1 (d)	1.81 dd (13.2, 4.2)	44.2 (d)
12	1.46 d (12.6)	51.0 (d)	1.25 d (12.6)	50.4 (d)	1.32 d (13.2)	51.8 (d)
13α	1.25 ddd (14.0, 14.0, 3.0)	25.9 (t)	1.23 ddd (14.0, 14.0, 3.0)	26.4 (t)	1.23 ddd (13.8, 13.8, 3.6)	26.3 (t)
13β	1.80 ddd (14.0, 3.0, 3.0, )	-	1.73 ddd (14.0, 3.0, 3.0)	-	1.72 ddd (13.8, 3.6, 3.6)	-
14α	1.66 ddd (14.0, 3.0, 3.0)	40.2 (t)	1.66 m	40.6 (t)	1.64 ddd (13.8, 3.6, 3.6)	41.1 (t)
14β	1.55 ddd (14.0, 14.0, 3.0)	-	1.59 m	-	1.46 ddd (13.8, 13.8, 3.6)	-
15	-	72.9 (s)	-	73.1 (s)	-	73.6 (s)
16	1.26 s	21.6 (q)	1.23 s	21.5 (q)	1.18 s	20.5 (q)
17	1.17 d (7.2)	21.3 (q)	1.05 d (6.6)	22.9 (q)	1.23 d (7.8)	20.0 (q)
18ax	1.01 s	24.2 (q)	0.93 s	18.3 (q)	0.99 s	25.7 (q)
19eq	1.03 s	25.1 (q)	1.02 s	19.4 (q)	1.04 s	25.2 (q)
20	1.05 s	22.0 (q)	1.29 s	20.9 (q)	0.98 s	19.8 (q)

***^a^*** ^1^H chemical shift values (d ppm from SiMe4) followed by multiplicity.

**Table 2:** Cytotoxicity of metabolites (**1**–**3**) against cancer cell lines.

Compounds	Cell line P388	Cell line HL-60	Cell line L1210
	IC_50_ (µM) *^a^*	IC_50_ (µM) *^a^*	IC_50_ (µM) *^a^*
**1**	7.9	6.8	7.6
**2**	51.9	59.7	85.2
**3**	80.1	78.9	134.1
5-fluorouracil *^b^*	6.1	5.1	4.5

***^a^*** DMSO was used as vehicle; *^b^* Positive control.

## Supplementary Materials

The following are available online at www.mdpi.com/1660-3397/15/6/169/s1. Table S1: Spectral data including 2D NMR data for **1**; Table S2: Spectral data including 2D NMR data for **2**; Table S3: Spectral data including 2D NMR data for **3**; Table S4: ^1^H NMR spectral data of MTPA esters **1a** and **1b** in CDCl_3_; Table S5: ^1^H NMR spectral data of MTPA esters **2a** and **2b** in CDCl_3_; Figure S1: ^1^H NMR spectra of **1** in CDCl_3_; Figure S2: ^13^C NMR spectra of **1** in CDCl_3_; Figure S3: ^1^H-^1^H COSY of **1**; Figure S4: NOESY of **1**; Figure S5: HMQC of **1**; Figure S6: HMBC of **1**; Figure S7: ^1^H NMR spectrum of **2** in CDCl_3_; Figure S8: ^13^C NMR spectrum of **2** in CDCl_3_; Figure S9: ^1^H-^1^H COSY of **2**; Figure S10: NOESY of **2**; Figure S11: HMQC of **2**; Figure S12: HMBC of **2**; Figure S13: ^1^H NMR spectrum of **3** in CDCl_3_; Figure S14: ^13^C NMR spectrum of **3** in CDCl_3_; Figure S15: ^1^H-^1^H COSY of **3**; Figure S16: NOESY of **3**; Figure S17: HMQC of **3**; Figure S18: HMBC of **3**; Figure S19: ^1^H NMR spectra of **1a** in CDCl_3_; Figure S20: ^1^H-^1^H COSY of **1a**; Figure S21: NOESY of **1a**; Figure S22: ^1^H NMR spectra of **1b** in CDCl_3_; Figure S23: ^1^H-^1^H COSY of **1b**; Figure S24: NOESY of **1b**; Figure S25: ^1^H NMR spectra of **2a** in CDCl_3_; Figure S26: ^1^H-^1^H COSY of **2a**; Figure S27: NOESY of **2a**; Figure S28: ^1^H NMR spectra of **2b** in CDCl_3_; Figure S29: ^1^H-^1^H COSY of **2b**; Figure S30: NOESY of **2b**.
