# Supplementary material for: Trichodermanins C–E, New Diterpenes with a Fused 6-5-6-6 Ring System Produced by a Marine Sponge-Derived Fungus
Source: Mar Drugs. 2017 Jun 9;15(6):169. doi: 10.3390/md15060169 (PMC5484119; doi:10.3390/md15060169)
Supplement: Supplementary file 1 [file marinedrugs-15-00169-s001.pdf]

# Supplementary Materials: Trichodermanins C–E, New Diterpenes with a Fused 6-5-6-6 Ring System Produced by a Marine Sponge-Derived Fungus

**Takeshi Yamada, Mayo Suzue, Takanobu Arai, Takashi Kikuchi and Reiko Tanaka**

**Scheme 1.** Structures of metabolites in the fungal strain ........ 2
**Table S1.** Spectral data including 2D NMR data for **1** ........ 2
**Table S2.** Spectral data including 2D NMR data for **2** ........ 3
**Table S3.** Spectral data including 2D NMR data for **3** ........ 4
**Table S4.** $^1$H NMR spectral data of MTPA esters **1a** and **1b** in $CDCl_3$ ........ 5
**Table S5.** $^1$H NMR spectral data of MTPA esters **2a** and **2b** in $CDCl_3$ ........ 6
**Figure S1.** $^1$H NMR spectrum of **1** in $CDCl_3$ ........ 7
**Figure S2.** $^{13}$C NMR spectrum of **1** in $CDCl_3$ ........ 8
**Figure S3.** $^1$H-$^1$H COSY of **1** ........ 9
**Figure S4.** NOESY of **1** ........ 10
**Figure S5.** HMQC of **1** ........ 11
**Figure S6.** HMBC of **1** ........ 12
**Figure S7.** $^1$H NMR spectrum of **2** in $CDCl_3$ ........ 13
**Figure S8.** $^{13}$C NMR spectrum of **2** in $CDCl_3$ ........ 14
**Figure S9.** $^1$H-$^1$H COSY of **2** ........ 15
**Figure S10.** NOESY of **2** ........ 16
**Figure S11.** HMQC of **2** ........ 17
**Figure S12.** HMBC of **2** ........ 18
**Figure S13.** $^1$H NMR spectrum of **3** in $CDCl_3$ ........ 19
**Figure S14.** $^{13}$C NMR spectrum of **3** in $CDCl_3$ ........ 20
**Figure S15.** $^1$H-$^1$H COSY of **3** ........ 21
**Figure S16.** NOESY of **3** ........ 22
**Figure S17.** HMQC of **3** ........ 23
**Figure S18.** HMBC of **3** ........ 24
**Figure S19.** $^1$H NMR spectra of **1a** in $CDCl_3$ ........ 25
**Figure S20.** $^1$H-$^1$H COSY of **1a** ........ 26
**Figure S21.** NOESY of **1a** ........ 27
**Figure S22.** $^1$H NMR spectra of **1b** in $CDCl_3$ ........ 28
**Figure S23.** $^1$H-$^1$H COSY of **1b** ........ 29
**Figure S24.** NOESY of **1b** ........ 30
**Figure S25.** $^1$H NMR spectra of **2a** in $CDCl_3$ ........ 31
**Figure S26.** $^1$H-$^1$H COSY of **2a** ........ 32
**Figure S27.** NOESY of **2a** ........ 33
**Figure S28.** $^1$H NMR spectra of **2b** in $CDCl_3$ ........ 34
**Figure S29.** $^1$H-$^1$H COSY of **2b** ........ 35
**Figure S30.** NOESY of **2b** ........ 36

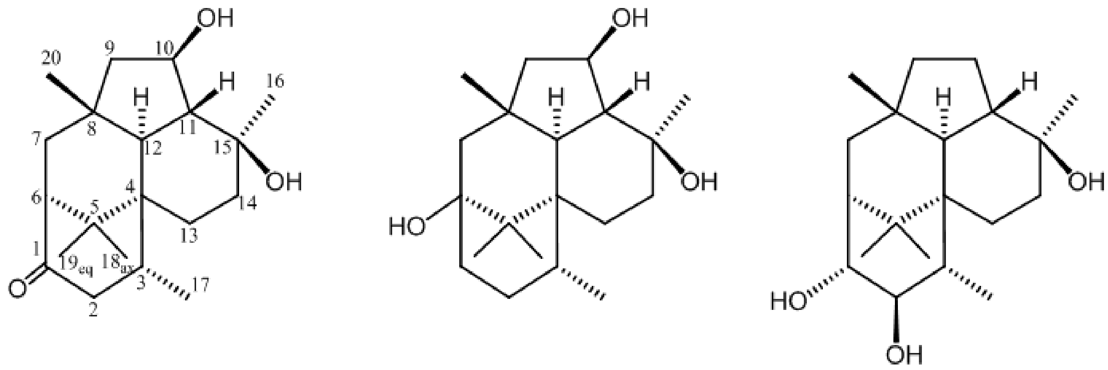

**1** **2** **3**

**Scheme 1.** Structures of metabolites in the fungal strain.

**Table S1.** Spectral data including 2D NMR data for **1**.

| Position | δH [a] | | *J*/Hz | $^1$H-$^1$H COSY | NOESY [b] | δC | HMBC (C) [c] | |
|---|---|---|---|---|---|---|---|---|
| 1 | - | - | - | - | - | 217.7 | 1 | - |
| 2α | 2.27 | dd | 20.4 (2β), 7.2 (3) | 2β, 3 | 17, 19 | 48.7 | 2α | 2.27 |
| 2β | 2.91 | dd | 20.4 (2α), 9.0 (3) | 2α, 3 | 20 | | 2β | 2.91 |
| 3 | 2.44 | dqd | 9.0 (2β), 7.2 (2α), 7.2 (17) | 2α, 2β, 17 | 11, 14β, 20 | 26.1 | 3 | 2.44 |
| 4 | - | - | - | - | - | 39.5 | 4 | - |
| 5 | - | - | - | - | - | 38.2 | 5 | - |
| 6 | 2.03 | dd | 3.6 (7α), 3.6 (7β) | 7α, 7β | 18, 19 | 58.0 | 6 | 2.03 |
| 7α | 1.76 | dd | 13.8 (7β), 3.6 (6) | 6, 7β | 9, 12, 18 | 41.4 | 7α | 1.76 |
| 7β | 1.92 | dd | 13.8 (7α), 3.6 (6) | 6, 7α | 9, 20 | - | 7β | 1.92 |
| 8 | - | | | | - | 39.0 | 8 | - |
| 9 | 1.50 | m | | 10 | 7α, 7β, 20 | 53.9 | 9 | 1.50 |
| 10 | 4.41 | ddd | 7.8 (9), 4.8 (11), 1.2 (9) | 9, 11 | 12, 16 | 72.6 | 10 | 4.41 |
| 11 | 1.95 | dd | 12.6 (12), 4.8 (10) | 10, 12 | 3, 20 | 54.7 | 11 | 1.95 |
| 12 | 1.46 | d | 12.6 (11) | 11 | 7α, 10, 16, 18 | 51.0 | 12 | 1.46 |
| 13α | 1.25 | ddd | 14.0 (13β), 14.0 (14β), 3.0 (14α) | 13β, 14α, 14β | 18 | 25.9 | 13α | 1.25 |
| 13β | 1.80 | ddd | 14.0 (13α), 3.0 (14β), 3.0 (14α) | 13α, 14α, 14β | 17, 19 | - | 13β | 1.80 |
| 14α | 1.66 | ddd | 14.0 (12β), 3.0 (13α), 3.0 (13β) | 13α, 13β, 14β | - | 40.2 | 14α | 1.66 |
| 14β | 1.55 | ddd | 14.0 (12α),14.0 (13α), 3.0 (13β) | 13α, 13β, 14α | 3, 17 | - | 14β | 1.55 |
| 15 | - | - | - | - | - | 72.9 | 15 | - |
| 16 | 1.26 | s | - | - | 10, 12 | 21.6 | 16 | 1.26 |
| 17 | 1.17 | d | 7.2 (3) | 3 | 2a, 13b, 14b, 19 | 21.3 | (q) | 2, 3, 4 |
| 18ax | 1.01 | s | - | - | 6, 7a, 12, 13a | 24.2 | (q) | 4, 5, 6, 19 |
| 19eq | 1.03 | s | - | - | 2a, 6, 13b, 17 | 25.1 | (q) | 4, 5, 6, 18 |
| 20 | 1.05 | s | - | - | 2b, 3, 7b, 9, 11 | 22.0 | (q) | 7, 8, 9, 12 |

[a] $^1$H chemical shift values (δ ppm from $SiMe_4$) followed by multiplicity and then the coupling constants (*J*/Hz). Figures in parentheses indicate the proton coupling with that position. [b] The correlations with geminal and vicinal protones are removed. [c] Long range $^1$H-$^{13}$C correlations from H to C observed in the HMBC experiment.

**Table S2.** Spectral data including 2D NMR data for **2**.

| Position | $\delta_H$ [a] | | *J*/Hz | $^1$H-$^1$H COSY | NOESY [b] | $\delta_C$ | | HMBC (C) [c] |
|---|---|---|---|---|---|---|---|---|
| 1α | 1.90 | ddd | 14.4 (1β), 2.4 (2α), 2.4 (2β) | 1β, 2α, 2β | 19 | 35.5 | (t) | 2, 6, 7 |
| 1β | 1.98 | ddd | 14.4 (2β), 10.8 (2α), 6.0 (2β) | 1α, 2α, 2β | 3, 20 | - | - | 2, 5, 6, 7 |
| 2α | 1.64 | m | - | 1α, 1β, 2β, 3 | 17, 19 | 29.5 | (t) | 1, 3, 17 |
| 2β | 2.12 | m | - | 1α, 1β, 2α, 3 | - | - | - | - |
| 3 | 2.14 | m | - | 2α, 2β | 1β, 11, 20 | 26.0 | (d) | 2, 5, 12, 17 |
| 4 | - | - | - | - | - | 41.0 | (s) | - |
| 5 | - | - | - | - | - | 44.1 | (s) | - |
| 6 | - | - | - | - | - | 74.9 | (s) | - |
| 7α | 1.56 | m | - | 7β | - | 51.2 | (t) | 1, 6, 8, 9, 20 |
| 7β | 1.62 | m | - | 7α | 20 | - | - | 1, 5, 6, 8, 12, 20 |
| 8 | - | - | - | - | - | 39.1 | (s) | |
| 9 | 1.51 | m | - | 10 | 20 | 54.4 | (t) | 7, 8, 20 |
| 10 | 4.39 | ddd | 8.4 (9), 4.8 (11), 1.2 (9) | 9, 11 | 12, 16 | 72.8 | (d) | 8, 15 |
| 11 | 1.88 | dd | 12.6 (12), 4.8 (10) | 10, 12 | 3, 20 | 55.1 | (d) | 10, 12, 15, 16 |
| 12 | 1.25 | d | 12.6 (11) | 11 | 10, 18 | 50.4 | (d) | 3, 4, 5, 8, 9, 10, 11, 13, 15, 20 |
| 13α | 1.23 | ddd | 14.0 (13β), 14.0 (14β), 3.0 (14α) | 13β, 14α, 14β | 18 | 26.4 | (t) | 14, 15 |
| 13β | 1.73 | ddd | 14.0 (13α), 3.0 (14β), 3.0 (14α) | 13α, 14α, 14β | 17, 19 | - | - | 14 |
| 14α | 1.66 | m | - | 13α, 13β, 14β | - | 40.6 | (t) | - |
| 14β | 1.59 | m | - | 13α, 13β, 14α | 17 | - | - | - |
| 15 | - | - | - | - | - | 73.1 | (s) | - |
| 16 | 1.23 | s | - | - | 10 | 21.5 | (q) | 11, 14, 15 |
| 17 | 1.05 | d | 6.6 (3) | 3 | 2α, 13β, 14β | 22.9 | (q) | 2, 3, 4 |
| 18ax | 0.93 | s | - | - | 12, 13α | 18.3 | (q) | 4, 5, 6, 19 |
| 19eq | 1.02 | s | - | - | 1α, 2α, 13β | 19.4 | (q) | 4, 5, 6, 18 |
| 20 | 1.29 | s | - | - | 1β, 3, 7β, 9, 11 | 20.9 | (q) | 7, 8, 9, 12 |

[a] $^1$H chemical shift values (δ ppm from $SiMe_4$) followed by multiplicity and then the coupling constants (*J*/Hz). Figures in parentheses indicate the proton coupling with that position. [b] The

correlations with geminal and vicinal protones are removed. [c] Long range $^{1}H$-$^{13}C$ correlations from H to C observed in the HMBC experiment.

**Table S3.** Spectral data including 2D NMR data for **3**.

| Position | $\delta_H$ [a] | | *J*/Hz | $^{1}H$-$^{1}H$ COSY | NOESY [b] | $\delta_C$ | | HMBC (C) [c] |
|---|---|---|---|---|---|---|---|---|
| 1 | 4.11 | d | 5.4 (2) | 2 | 7β, 20 | 80.4 | (d) | 2, 5, 6, 7 |
| 2 | 3.88 | dd | 7.8 (3), 5.4 (1) | 1, 3 | 17, 19 | 83.7 | (d) | 1, 3, 17 |
| 3 | 1.88 | qd | 7.8 (17), 7.8 (3) | 2α, 2β, 17 | 11, 20 | 36.6 | (d) | 2, 4, 5, 12, 17 |
| 4 | - | - | - | - | - | 41.2 | (s) | - |
| 5 | - | - | - | - | - | 39.4 | (s) | - |
| 6 | 1.50 | dd | 4.8 (7α), 3.0 (7β) | 7α, 7β | 18, 19 | 53.2 | (d) | 4 |
| 7α | 1.78 | dd | 13.8 (7β), 4.8 (6) | 6, 7β | 9α, 12, 18 | 40.9 | (t) | 1, 6, 8, 9, 20 |
| 7β | 1.70 | dd | 13.8 (7α), 3.0 (6) | 6, 7α | 1, 20 | - | - | 5, 12 |
| 8 | - | - | - | - | - | 39.6 | (s) | - |
| 9α | 1.03 | m | - | 9β, 10α, 10β | 7α, 12 | 43.5 | (t) | - |
| 9β | 1.43 | m | - | 9α, 10α, 10β | 20 | - | - | 20 |
| 10α | 1.59 | m | - | 9α, 9β, 10β, 11 | 16 | 21.6 | (t) | 8, 15 |
| 10β | 1.80 | m | - | 9α, 9β, 10α, 11 | - | - | - | - |
| 11 | 1.81 | dd | 13.2 (12), 4.2 (10) | 10α, 10β, 12 | 3, 14β, 20 | 44.2 | (d) | 10, 12, 15, 16 |
| 12 | 1.32 | d | 13.2 (11) | 11 | 7α, 9α, 16, 18 | 51.8 | (d) | 3, 4, 5, 8, 11, 15, 20 |
| 13α | 1.23 | ddd | 13.8 (13β), 13.8 (14β), 3.6 (14α) | 13β, 14α, 14β | 18 | 26.3 | (t) | - |
| 13β | 1.72 | ddd | 13.8 (13α), 3.6 (14β), 3.6 (14α) | 13α, 14α, 14β | 17, 19 | - | - | 5, 12 |
| 14α | 1.64 | ddd | 13.8 (12β), 3.6 (13α), 3.6 (13β) | 13α, 13β, 14β | 16 | 41.1 | (t) | - |
| 14β | 1.46 | ddd | 13.8 (12α), 13.8 (13α), 3.6 (13β) | 13α, 13β, 14α | 11, 17 | - | - | 11 |
| 15 | - | - | - | - | - | 73.6 | (s) | - |
| 16 | 1.18 | s | - | - | 10α, 12, 14α | 20.5 | (q) | 11, 14, 15 |
| 17 | 1.23 | d | 7.2 (3) | 3 | 2, 13β, 14β, 19 | 20.0 | (q) | 2, 3, 4 |
| 18αx | 0.99 | s | - | - | 6, 7α, 12, 13α | 25.7 | (q) | 4, 5, 6, 19 |
| 19eq | 1.04 | s | - | - | 2, 6, 13β, 17 | 25.2 | (q) | 4, 5, 6, 18 |
| 20 | 0.98 | s | - | - | 1, 3, 7β, 9β, 11 | 19.8 | (q) | 7, 8, 9, 12 |

[a] $^{1}H$ chemical shift values ($\delta$ ppm from $SiMe_4$) followed by multiplicity and then the coupling constants (*J*/Hz). Figures in parentheses indicate the proton coupling with that position. [b] The correlations with geminal and vicinal protones are removed. [c] Long range $^{1}H$-$^{13}C$ correlations from H to C observed in the HMBC experiment.

**Table S4.** $^{1}$H NMR spectral data of MTPA esters **1a** and **1b** in $CDCl_3$.

| Position | $\delta_H$[a] | | *J*/Hz | $\delta_H$[a] | | *J*/Hz |
|---|---|---|---|---|---|---|
| | **1a** | | | **1b** | | |
| 1 | - | - | - | - | - | - |
| 2α | 2.26 | dd | 19.8 (2β), 7.2 (3) | 2.26 | dd | 20.4 (2β), 7.8 (3) |
| 2β | 2.87 | dd | 19.8 (2α), 7.8 (3) | 2.87 | dd | 20.4 (2α), 9.0 (3) |
| 3 | 2.30 | m | - | 2.32 | m | - |
| 4 | - | - | - | - | - | - |
| 5 | - | - | - | - | - | - |
| 6 | 2.04 | dd | 4.2 (7α), 3.0 (7β) | 2.03 | dd | 4.8 (7α), 3.6 (7β) |
| 7α | 1.78 | m | | 1.76 | dd | 13.8 (7β), 4.8 (6) |
| 7β | 1.96 | dd | 13.8 (7α), 3.0 (6) | 1.92 | dd | 13.8 (7α), 3.6 (6) |
| 8 | - | - | - | - | - | - |
| 9 | 1.60 | m | - | 1.55 | m | - |
| 10 | 5.41 | dd | 7.8 (9), 4.8 (11) | 5.41 | dd | 7.2 (9), 4.8 (11) |
| 11 | 2.00 | dd | 14.4 (12), 4.8 (10) | 2.09 | dd | 14.4 (12), 4.8 (10) |
| 12 | 1.53 | d | 14.4 (11) | 1.51 | d | 14.4 (11) |
| 13α | 1.27 | ddd | 14.4 (13β), 14.4 (14β), 3.6 (14α) | 1.28 | ddd | 14.4 (13β), 14.4 (14β), 3.6 (14α) |
| 13β | 1.8 | ddd | 14.4 (13α), 3.6 (14β), 3.6 (14α) | 1.81 | ddd | 14.4 (13α), 3.6 (14β), 3.6 (14α) |
| 14α | 1.69 | ddd | 13.2 (14β), 3.6 (13α), 3.6 (13β) | 1.70 | ddd | 13.2 (14β), 3.6 (13α), 3.6 (13β) |
| 14β | 1.52 | m | - | 1.54 | m | - |
| 15 | - | - | - | - | - | - |
| 16 | 1.34 | s | - | 1.34 | s | - |
| 17 | 1.16 | d | 7.2 (3) | 1.16 | d | 7.2 (3) |
| 18ax | 1.02 | s | - | 1.02 | s | - |
| 19eq | 1.05 | s | - | 1.04 | s | - |
| 20 | 0.87 | s | - | 0.79 | s | - |
| $OCH_3$ | 3.54 | s | - | 3.57 | s | - |
| Ar.H | 7.38-7.43 | m | - | 7.38-7.43 | m | - |
| Ar.H | 7.49-7.57 | m | - | 7.52-7.61 | m | - |

[a] [1]H chemical shift values (δ ppm from $SiMe_4$) followed by multiplicity and then the coupling constants (*J*/Hz). Figures in parentheses indicate the proton coupling with that position.

**Table S5.** $^1$H NMR spectral data of MTPA esters **2a** and **2b** in $CDCl_3$.

| **Position** | $\delta_H$ $^a$ | | ***J*/Hz** | $\delta_H$ $^a$ | | ***J*/Hz** | |
|---|---|---|---|---|---|---|---|
| | **2a** | | | **2b** | | | |
| 1α | 1.89 | m | - | 1.89 | m | - | |
| 1β | 1.97 | m | - | 1.97 | m | - | |
| 2α | 1.64 | m | - | 1.64 | m | - | |
| 2β | 2.10 | m | - | 2.10 | m | - | |
| 3 | 2.01 | m | - | 2.02 | m | - | |
| 4 | - | - | - | - | - | - | |
| 5 | - | - | - | - | - | - | |
| 6 | - | - | - | - | - | - | |
| 7α | 1.60 | m | - | 1.57 | m | - | |
| 7β | 1.74 | d | 13.8 (7α) | 1.64 | m | - | |
| 8 | - | - | - | - | - | - | |
| 9 | 1.60 | m | - | 1.55 | m | - | |
| 10 | 5.41 | dd | 8.4 (9), 4.8 (11) | 5.41 | dd | 8.4 (9), 4.8 (11) | |
| 11 | 1.96 | dd | 14.4 (12), 4.8 (10) | 2.04 | dd | 14.4 (12), 4.8 (10) | |
| 12 | 1.33 | d | 14.4 (11) | 1.31 | d | 14.4 (11) | |
| 13α | 1.25 | ddd | 14.4 (13β), 14.4 (14β), 3.6 (14α) | 1.26 | ddd | 14.4 (13β), 14.4 (14β), 3.6 (14α) | |
| 13β | 1.73 | ddd | 14.4 (13α), 3.6 (14α), 3.6 (14β) | 1.74 | ddd | 14.4 (13α), 3.6 (14α), 3.6 (14β) | |
| 14α | 1.60 | ddd | 14.4 (14β), 3.6 (13α), 3.6 (13β) | 1.61 | m | - | |
| 14β | 1.48 | ddd | 14.4 (13α), 14.4 (14α), 3.6 (13β) | 1.50 | ddd | 14.4 (13α), 14.4 (14α), 3.6 (13β) | |
| 15 | - | - | - | - | - | - | - |
| 16 | 1.31 | s | - | 1.31 | s | - | - |
| 17 | 1.04 | d | 7.2 (3) | 1.04 | d | 7.2 (3) | - |
| 18ax | 0.95 | s | - | 0.94 | s | - | - |
| 19eq | 1.03 | s | - | 1.02 | - | - | - |
| 20 | 1.14 | s | - | 1.06 | - | - | - |
| $OCH_3$ | 3.56 | s | - | 3.56 | s | - | - |
| Ar.H | 7.26-7.42 | m | - | 7.26-7.41 | m | - | - |
| Ar.H | 7.52 | m | - | 7.55 | m | - | - |

$^a$ $^1$H chemical shift values (δ ppm from $SiMe_4$) followed by multiplicity and then the coupling constants (*J*/Hz). Figures in parentheses indicate the proton coupling with that position.

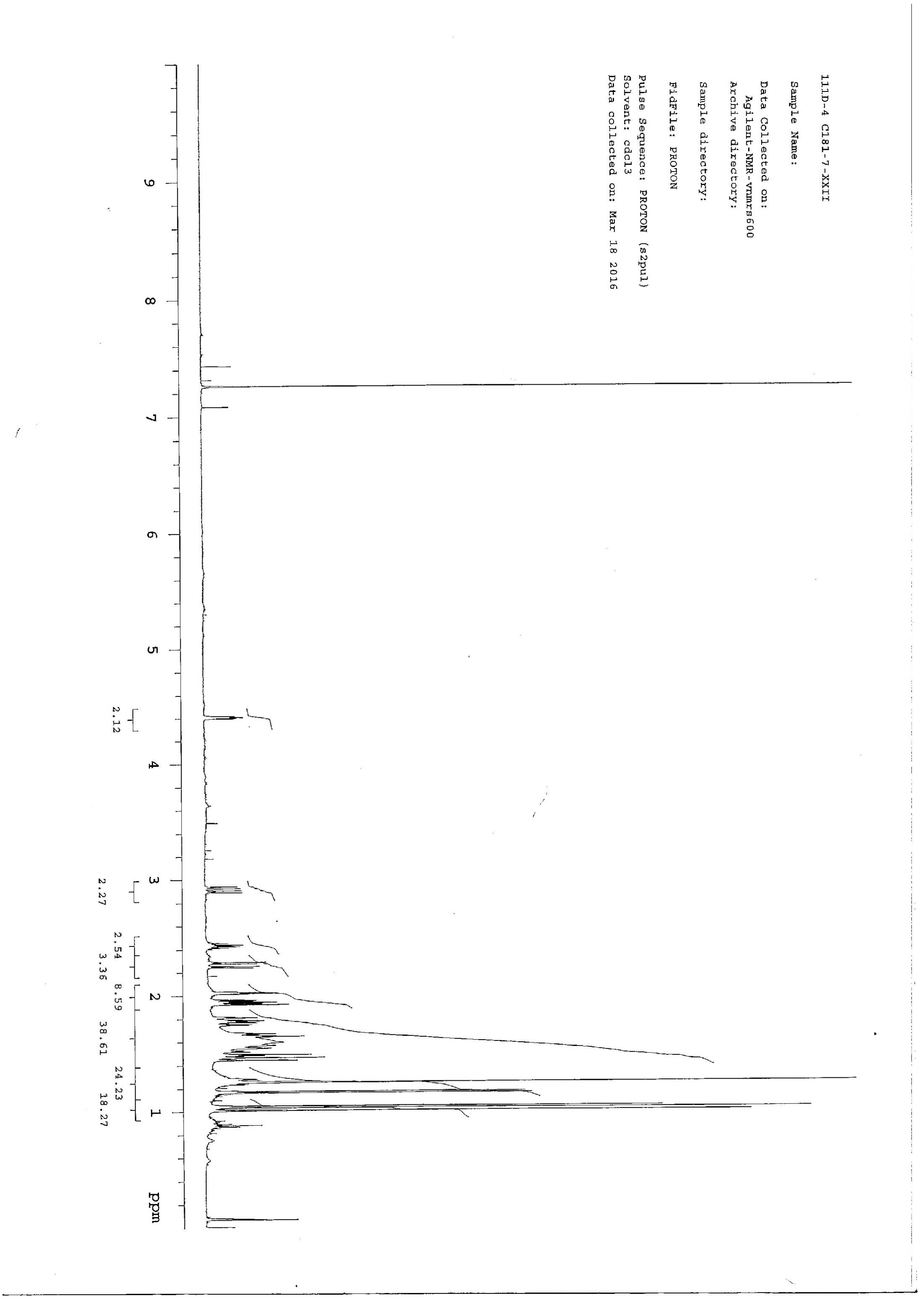


**Figure S1.** $^1$H NMR spectrum of **1**.

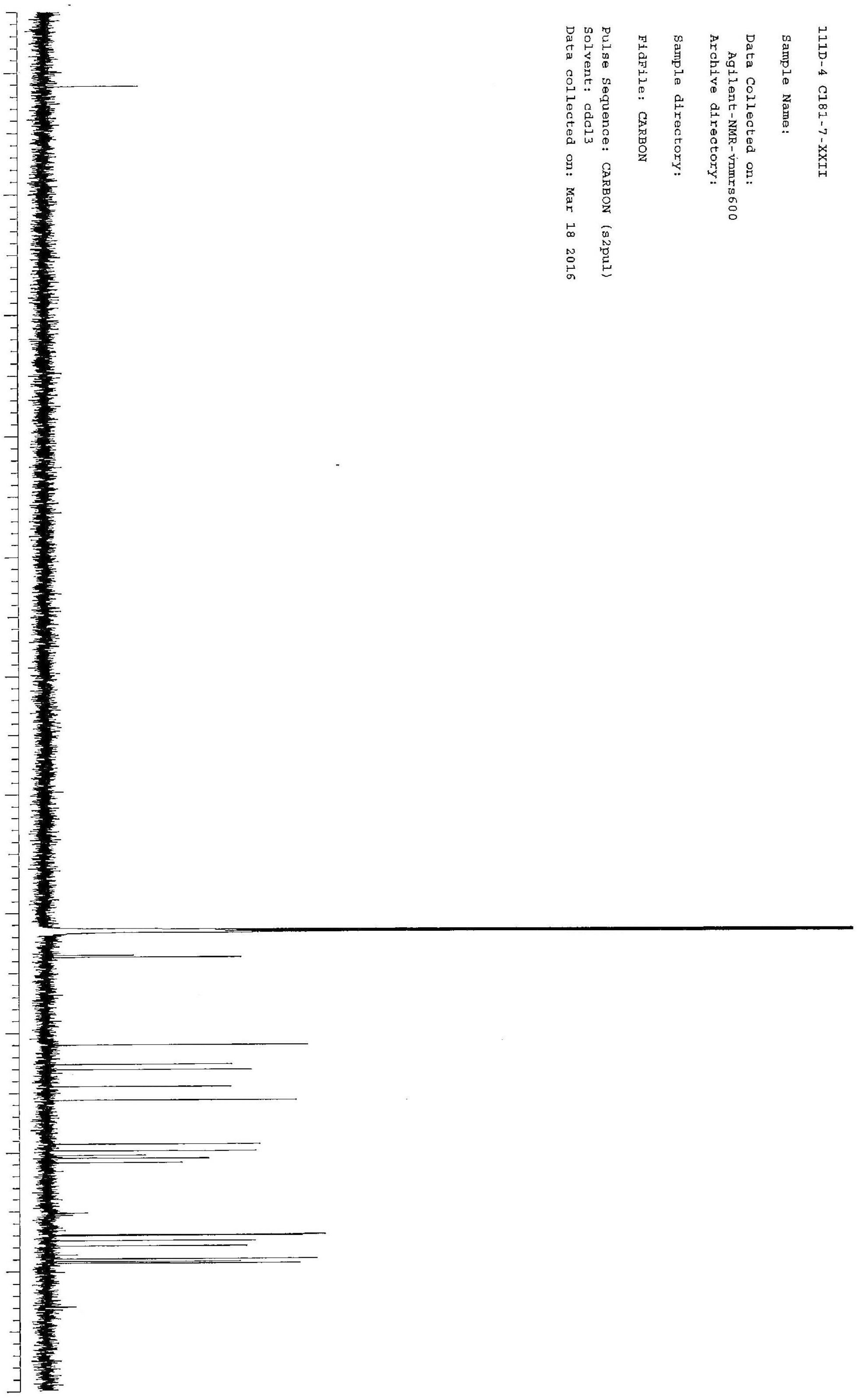


**Figure S2.** $^{13}$C NMR spectrum of **1** in $CDCl_3$.

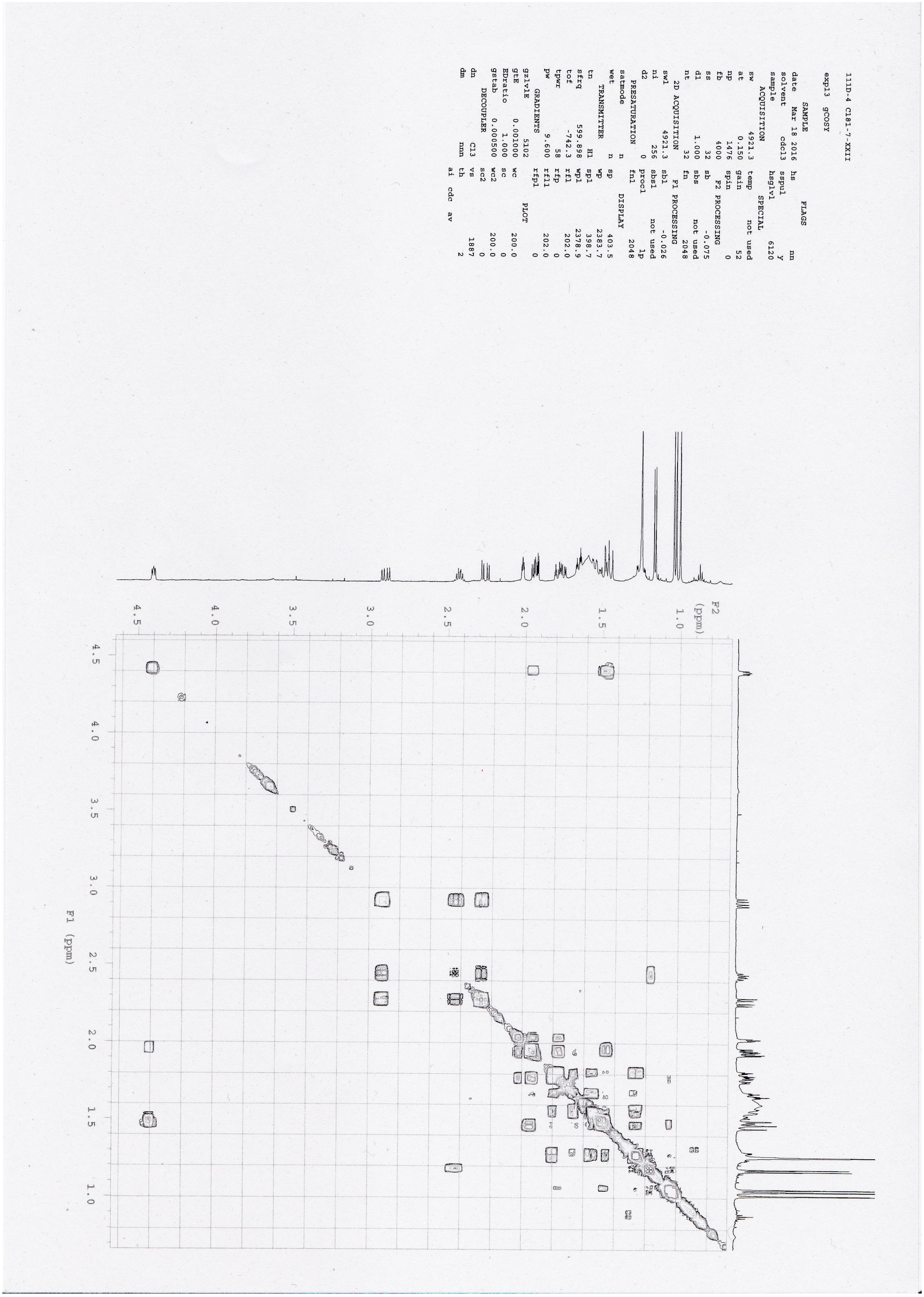

**Figure S3.** $^{1}$H-$^{1}$H COSY of **1**.

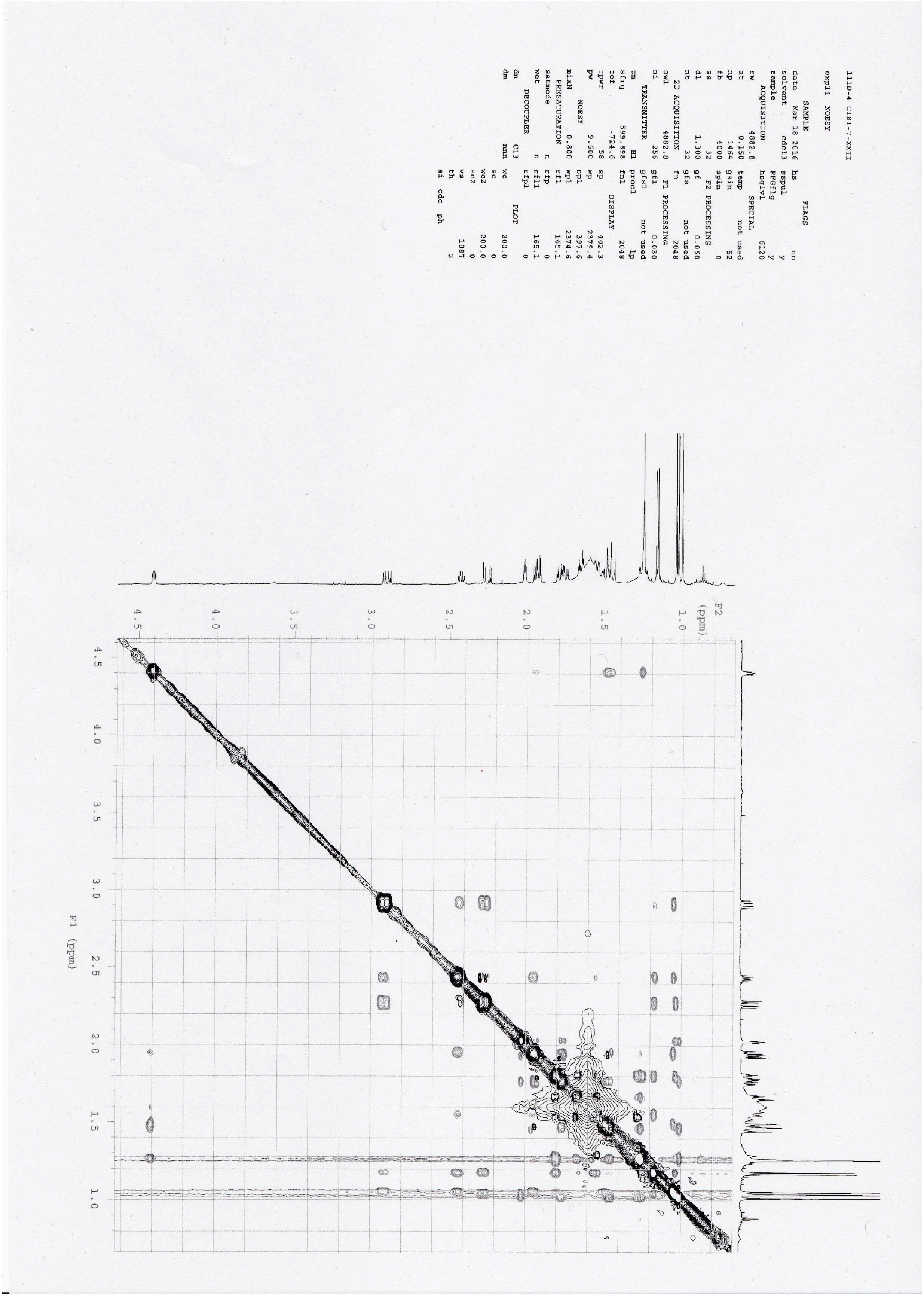

Figure S4. NOESY of 1.

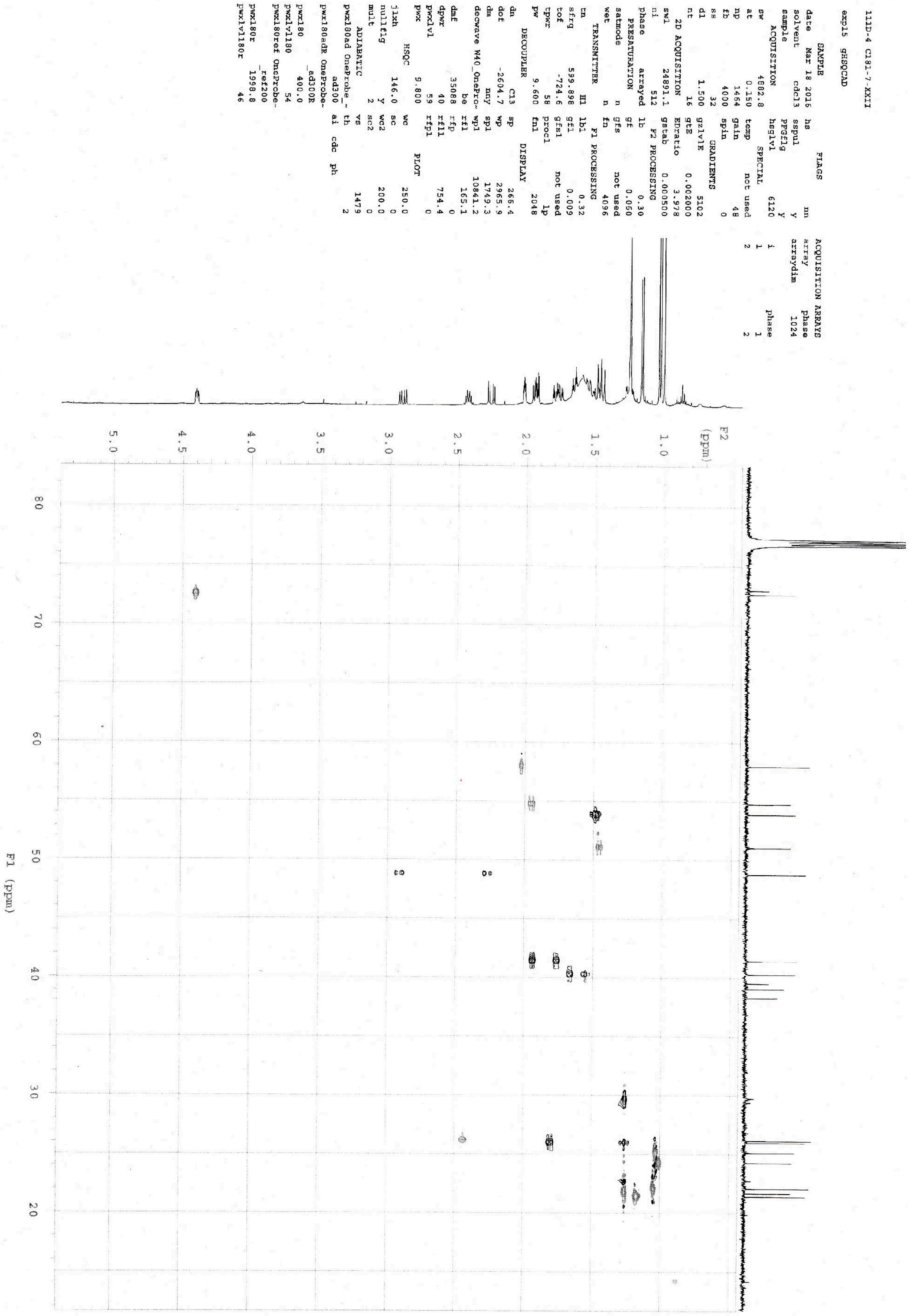


**Figure S5.** HMQC of **1**.

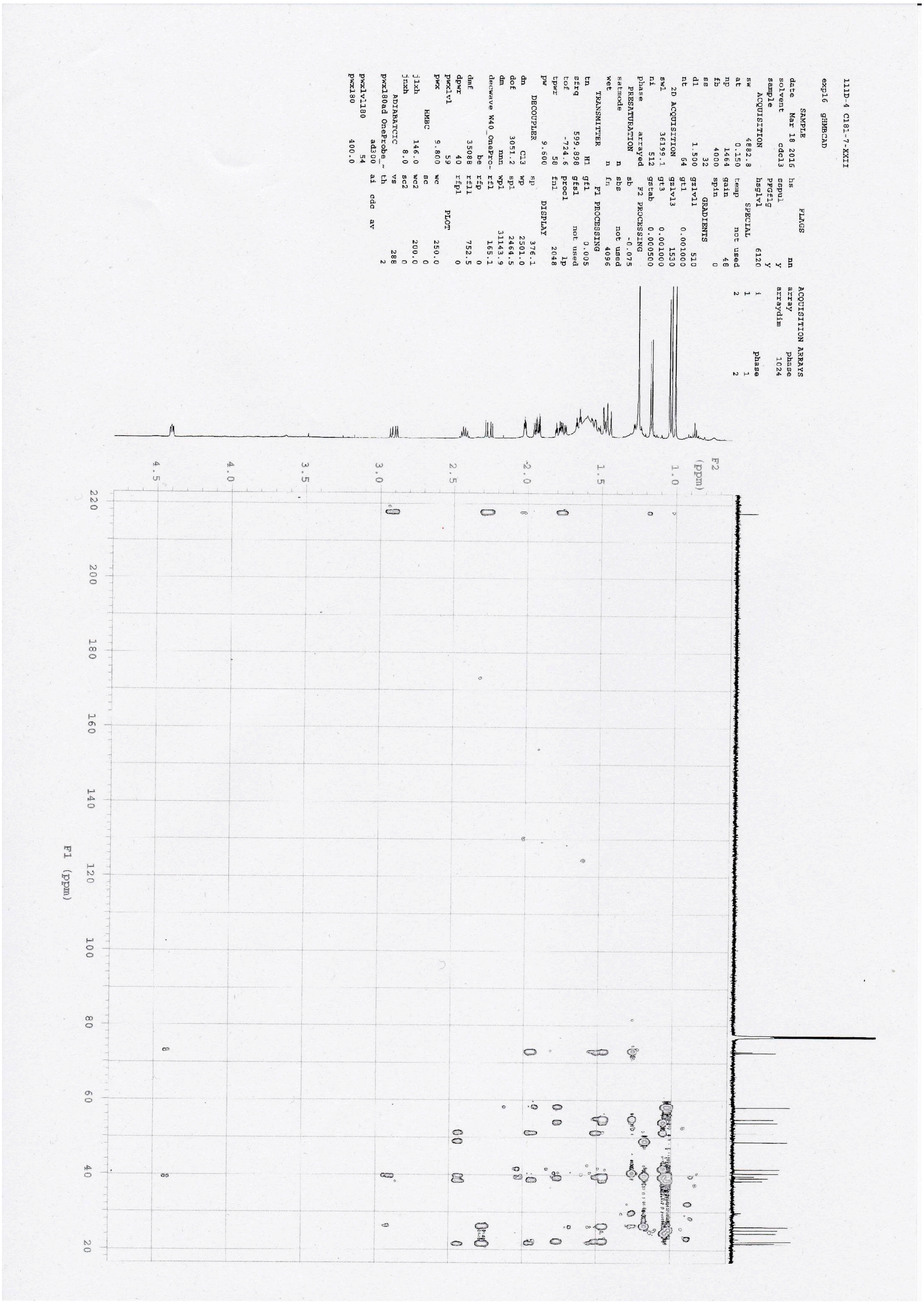

**Figure S6.** HMBC of 1.

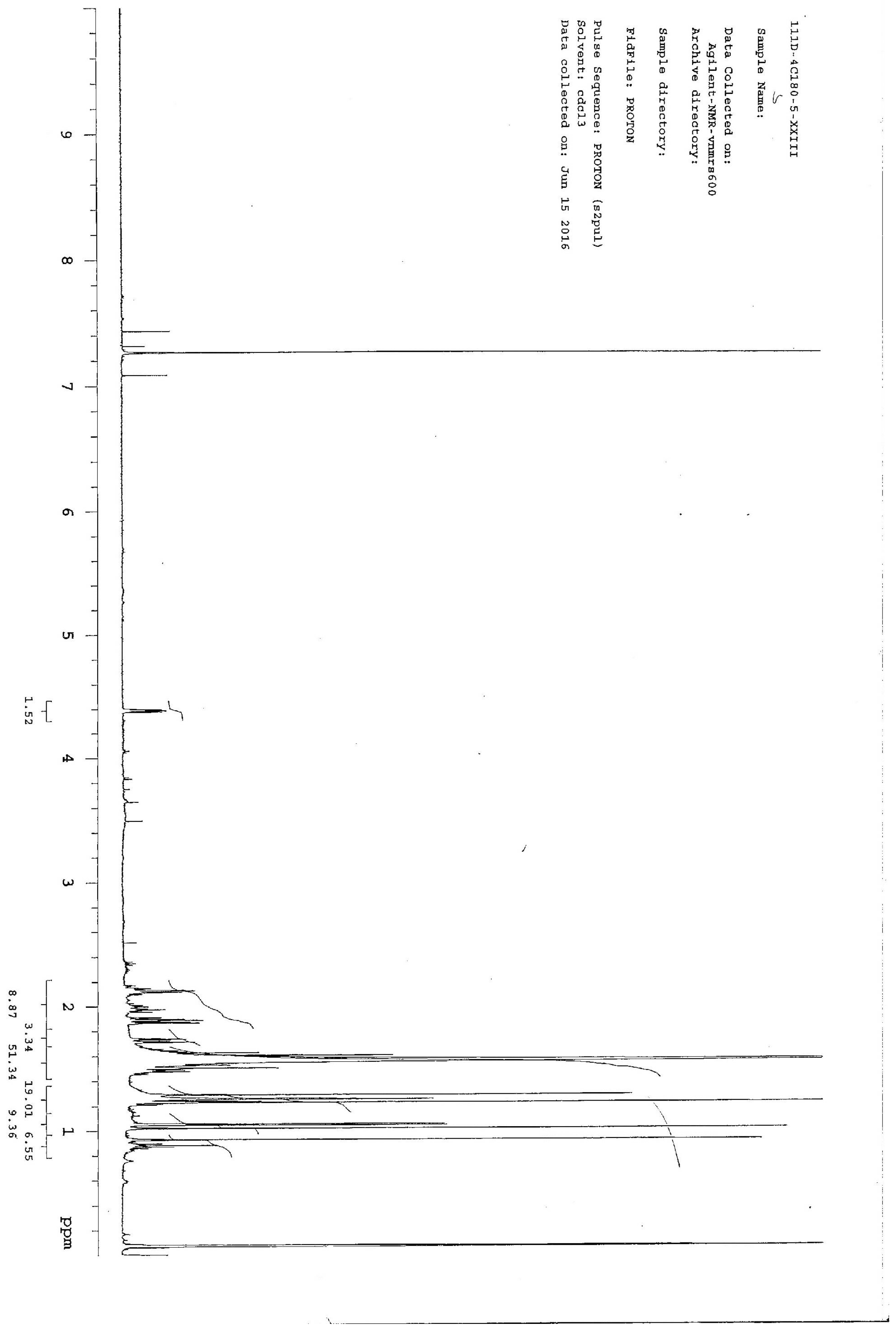


**Figure S7.** $^{1}H$ NMR pectrum of **2** in $CDCl_3$.

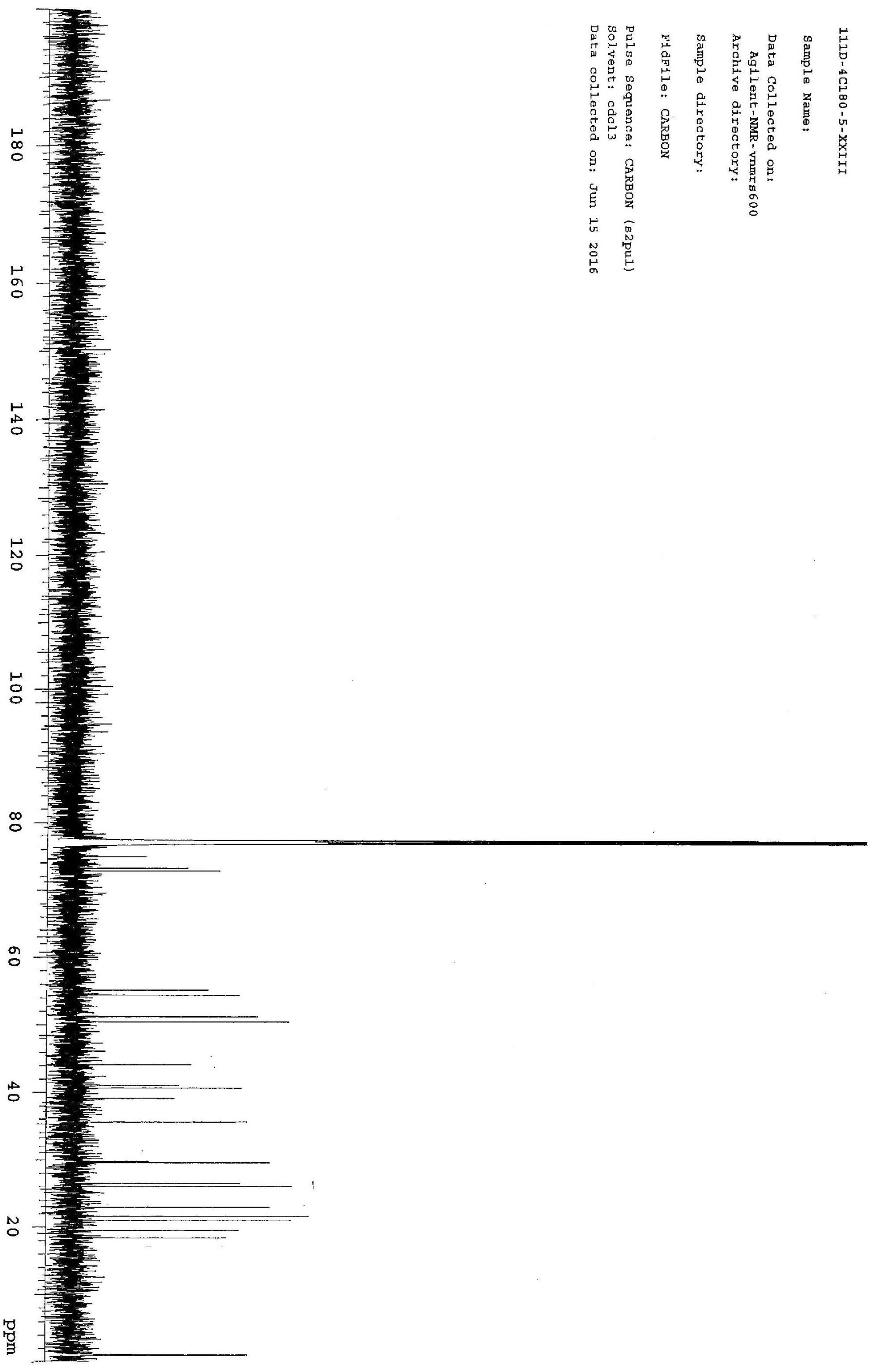


Figure S8. $^{13}C$ NMR spectrum of 2 in $CDCl_3$.

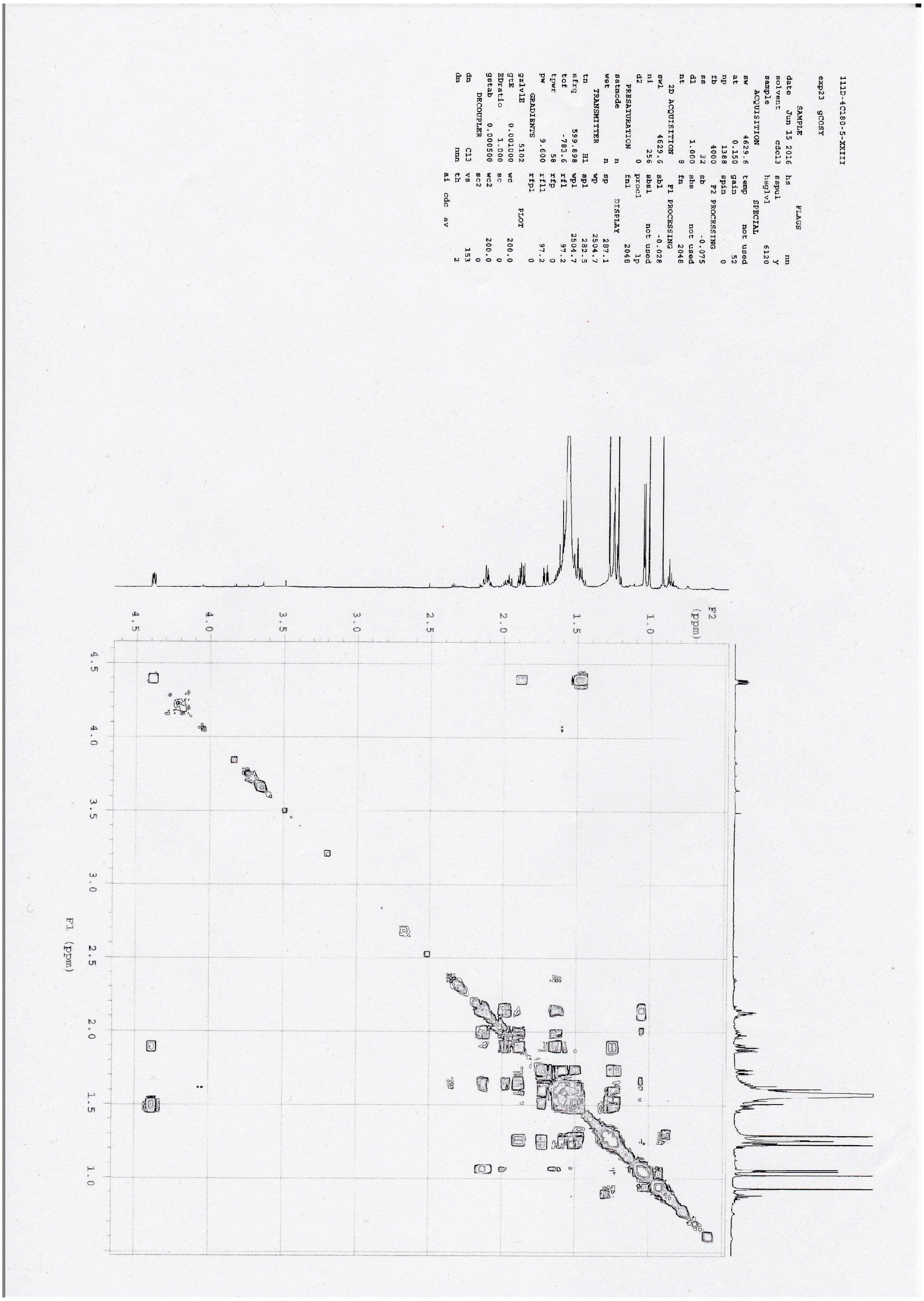

**Figure S9.** $^1H$-$^1H$ COSY of **2**.

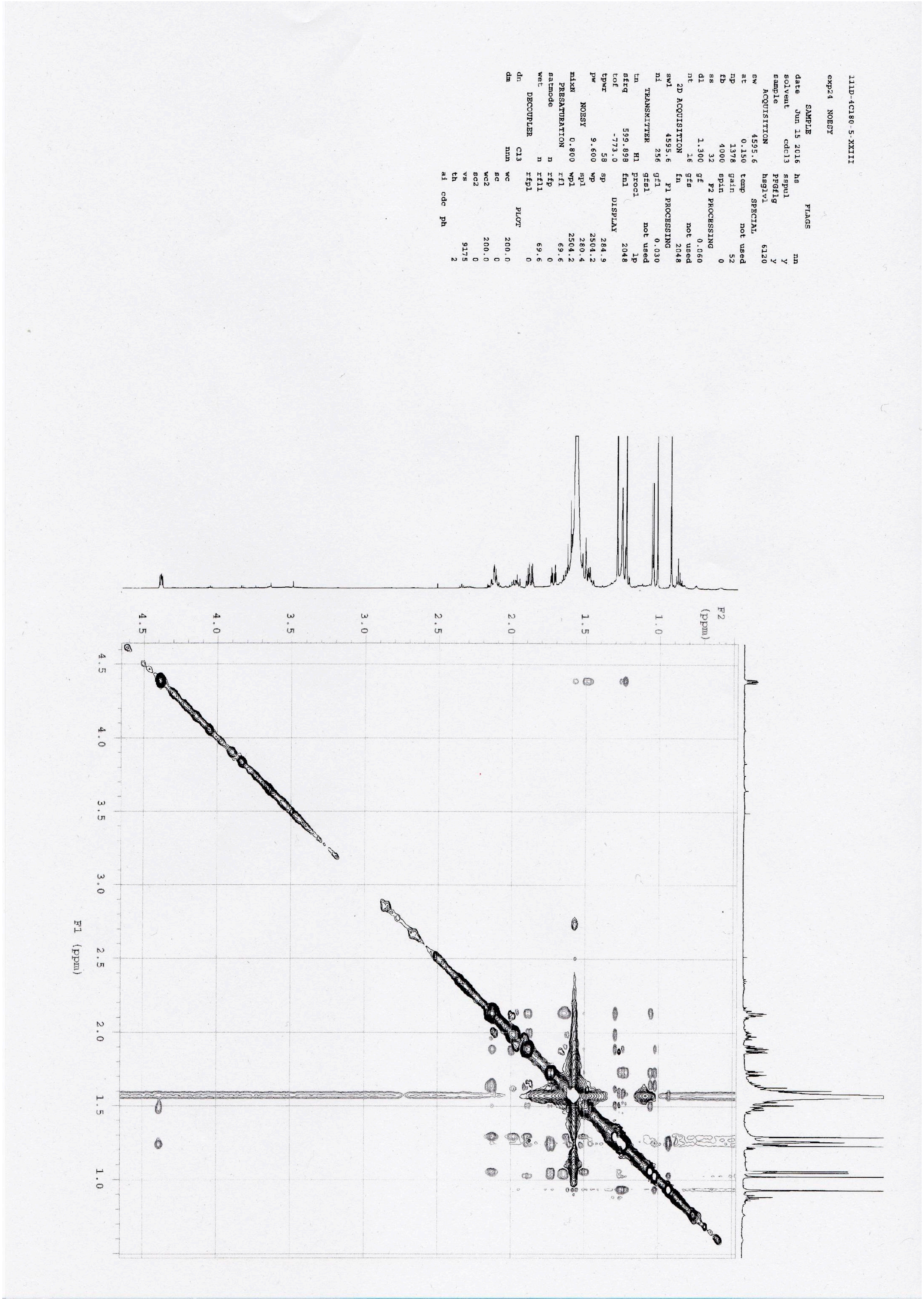


**Figure S10.** NOESY of **2**.

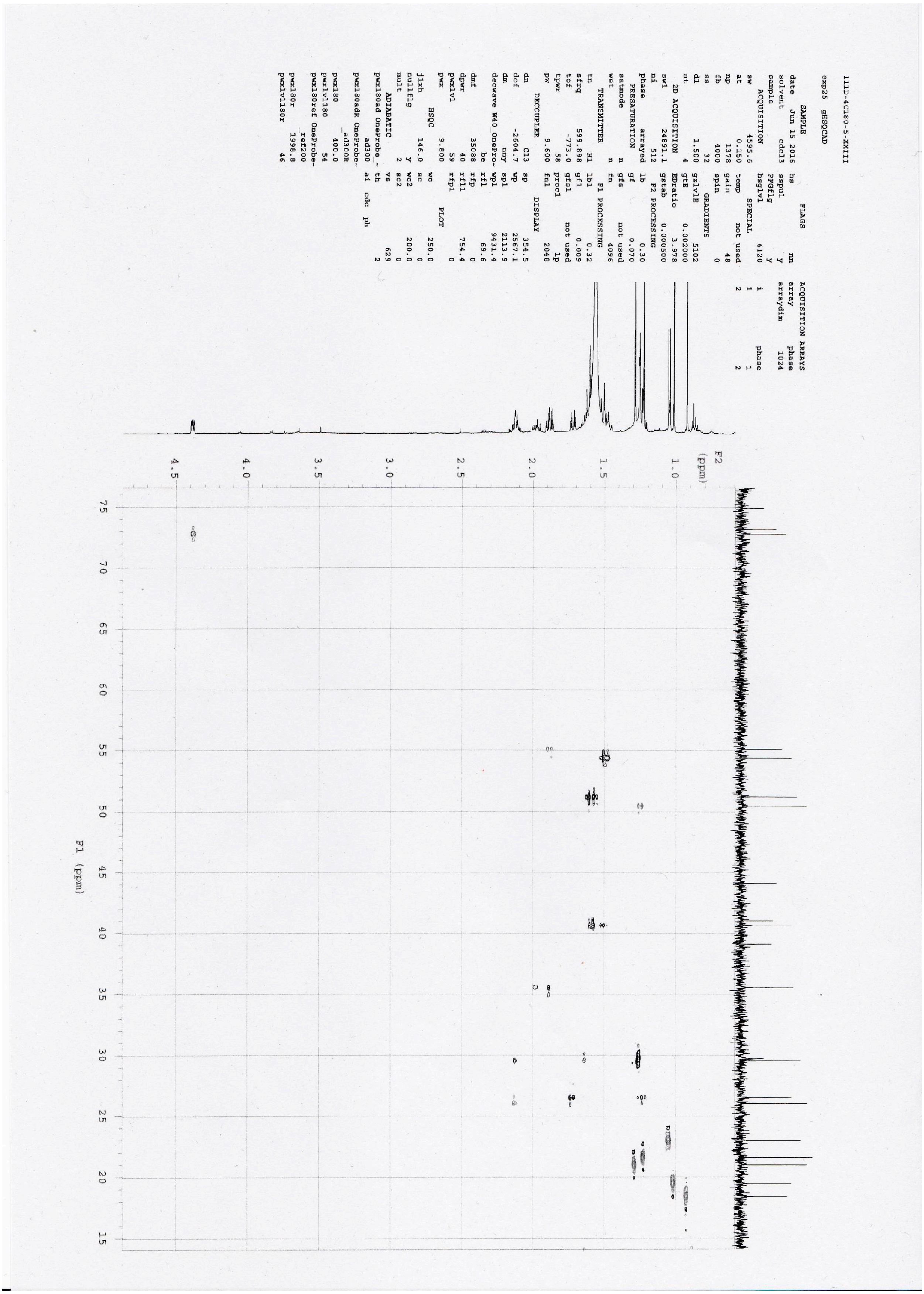

**Figure S11.** HMQC of **2**.

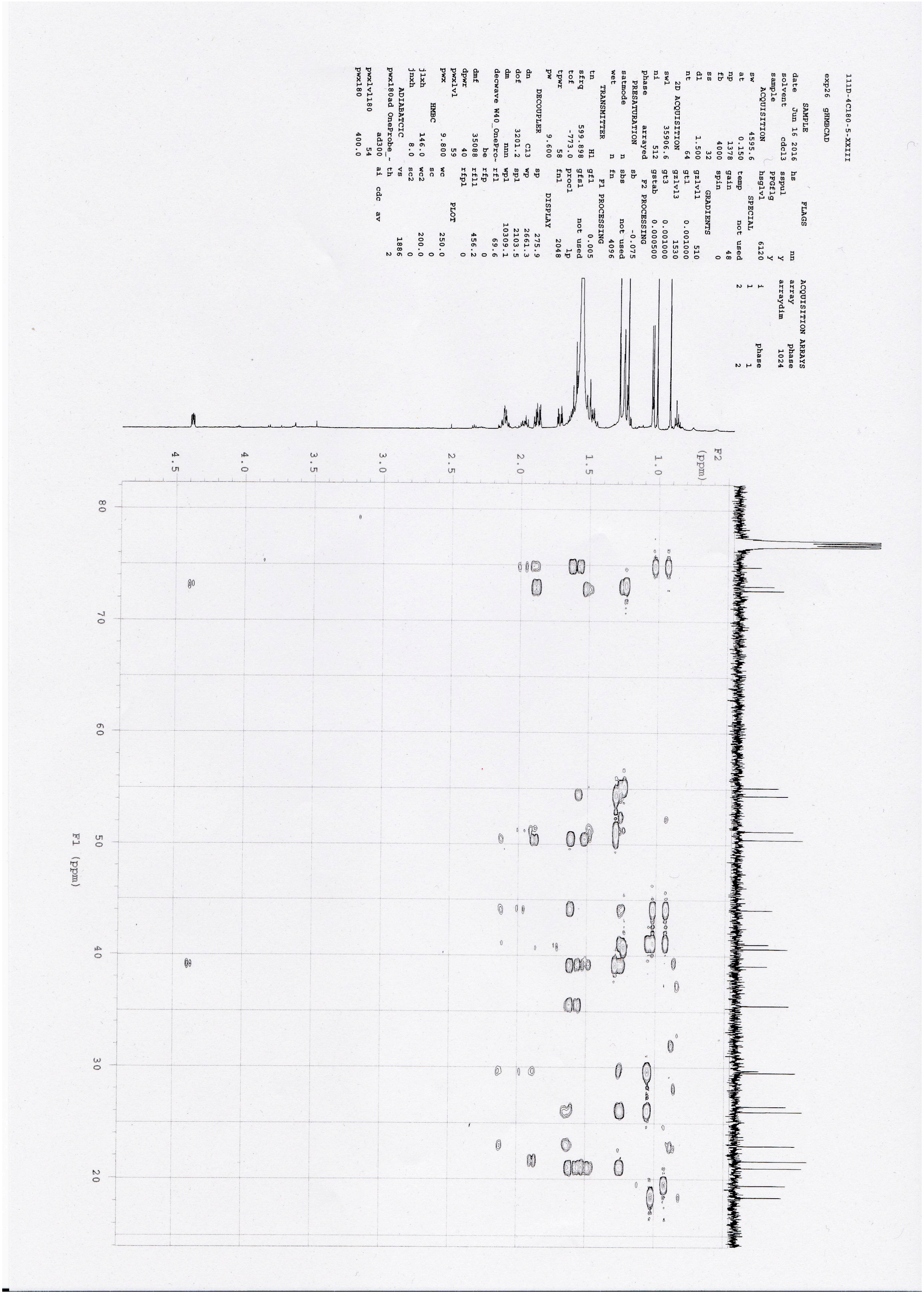

**Figure S12.** HMBC of **2**.

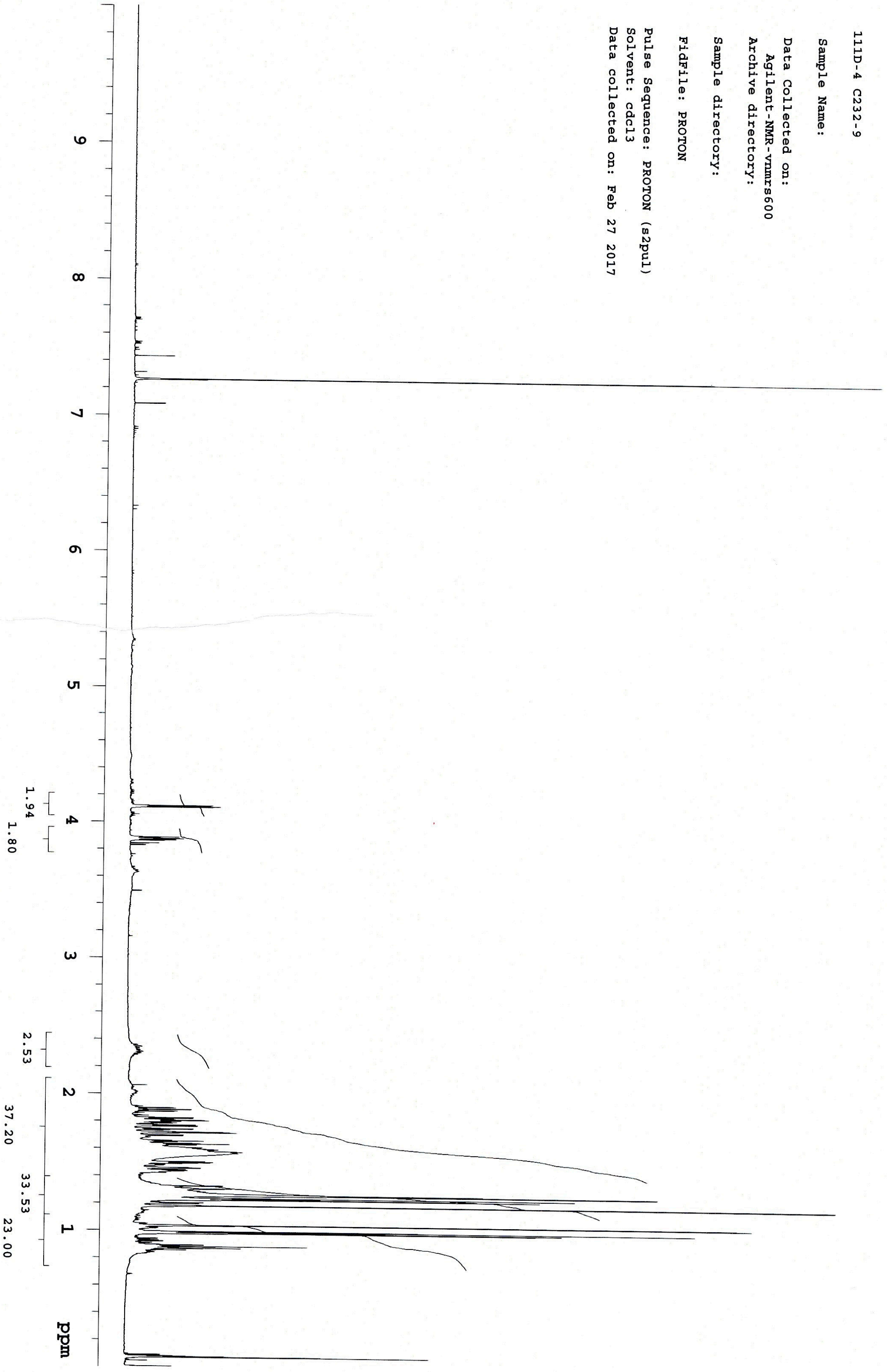


**Figure S13.** $^{1}H$ NMR spectrum of **3** in $CDCl_3$.

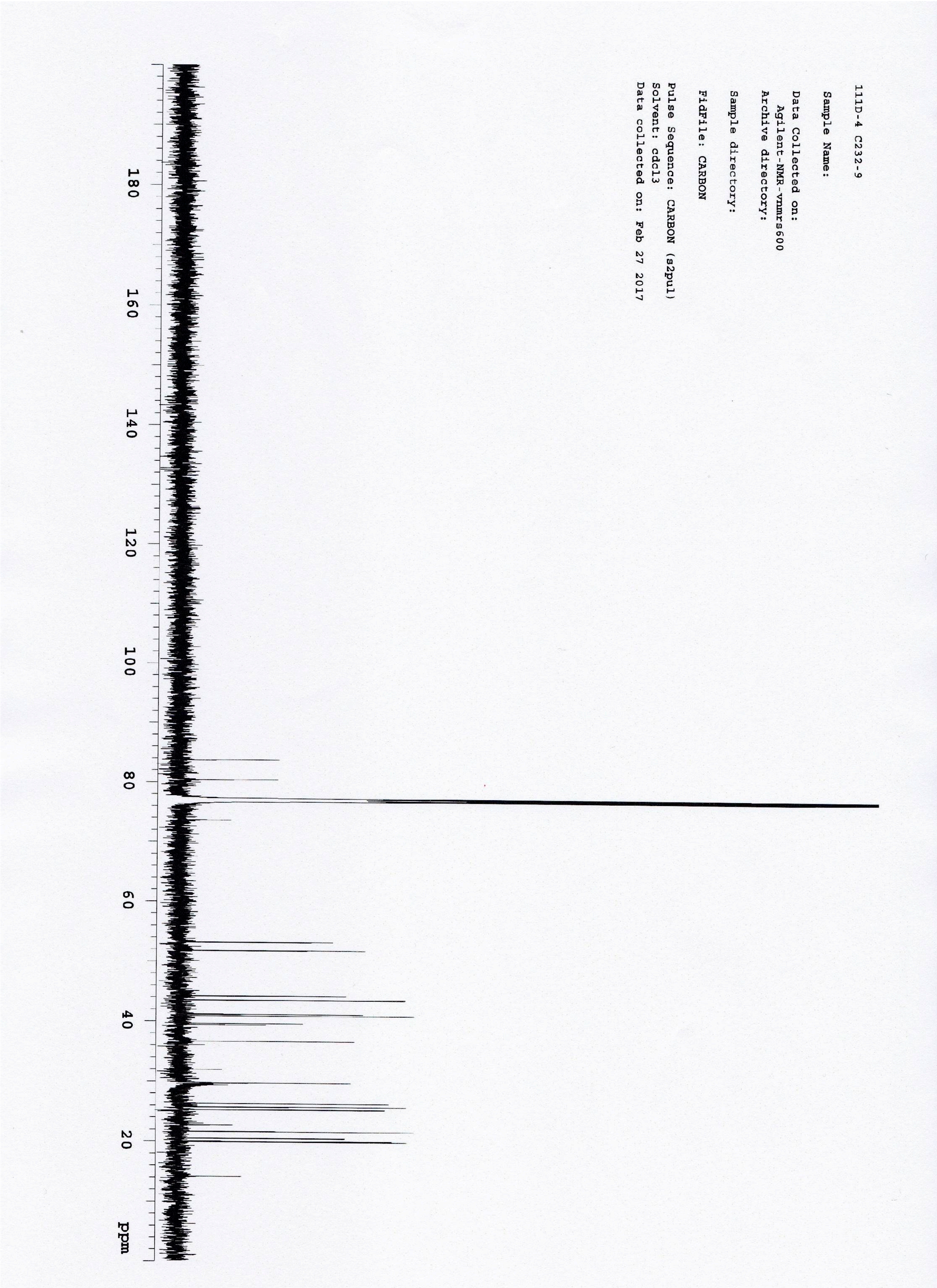


**Figure S14.** $^{13}$C NMR spectrum of **3** in $CDCl_3$.

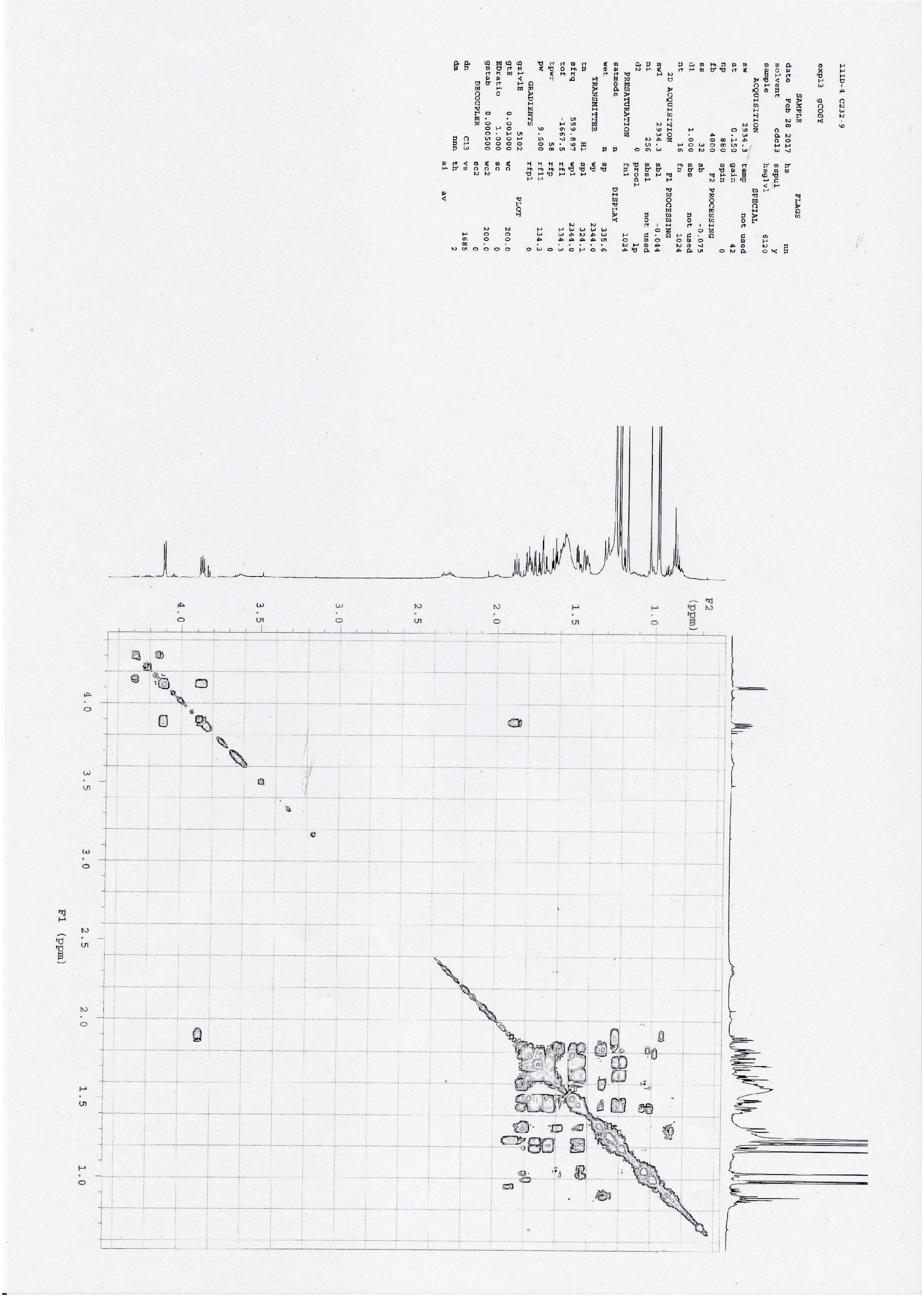

**Figure S15.** $^{1}H$-$^{1}H$ COSY of **3**.

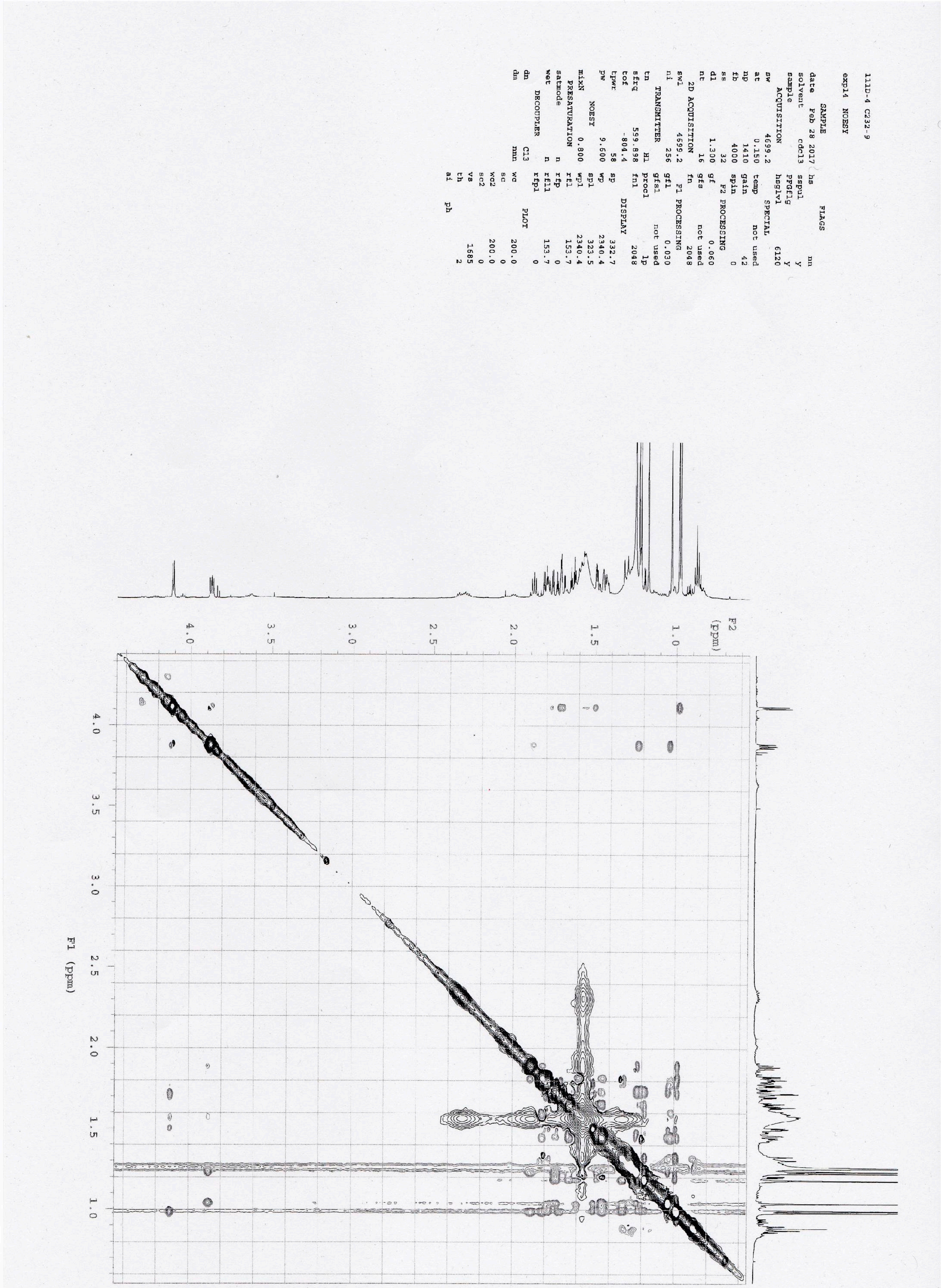


**Figure S16.** NOESY of **3**.

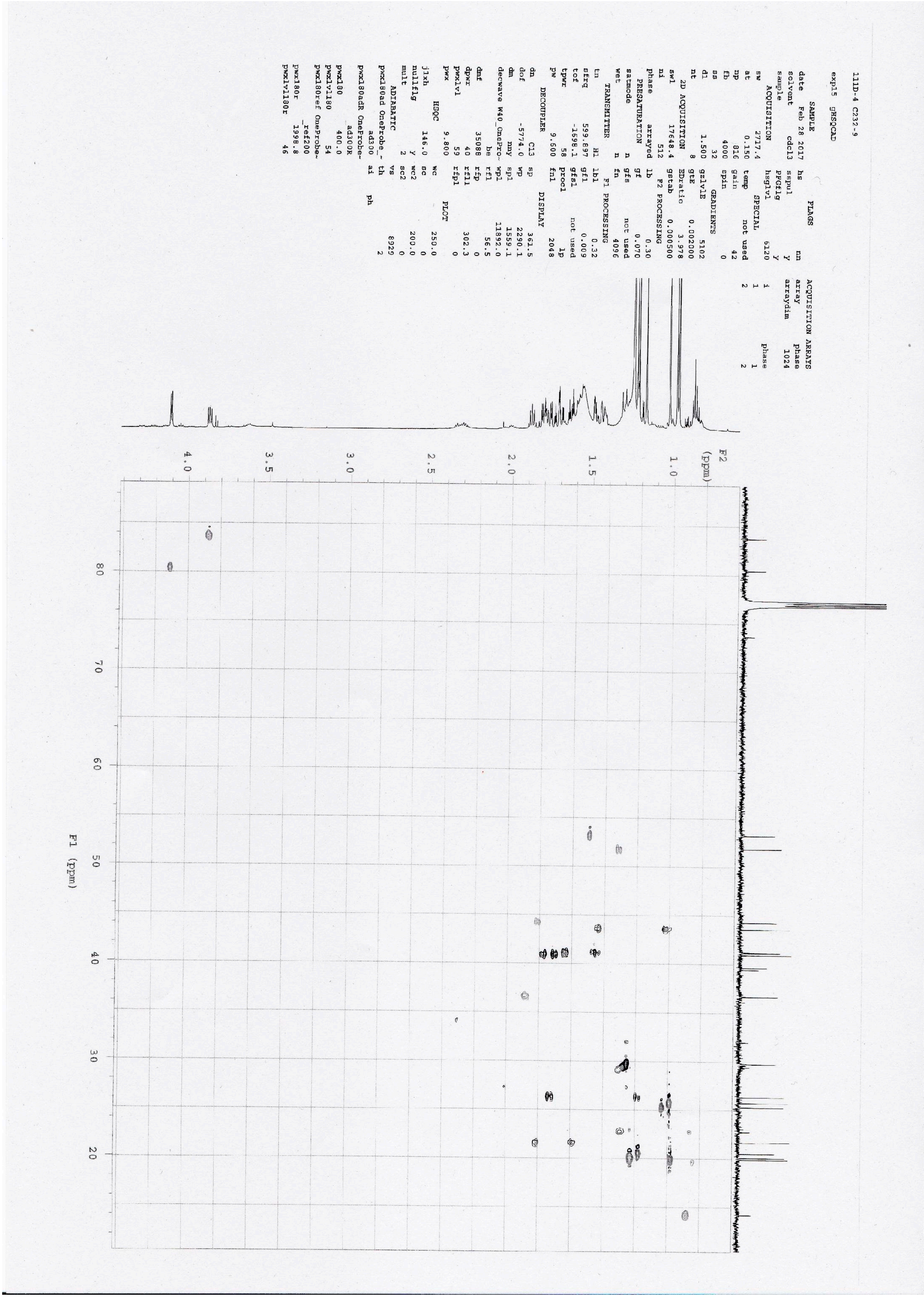


**Figure S17.** HMQC of **3**.

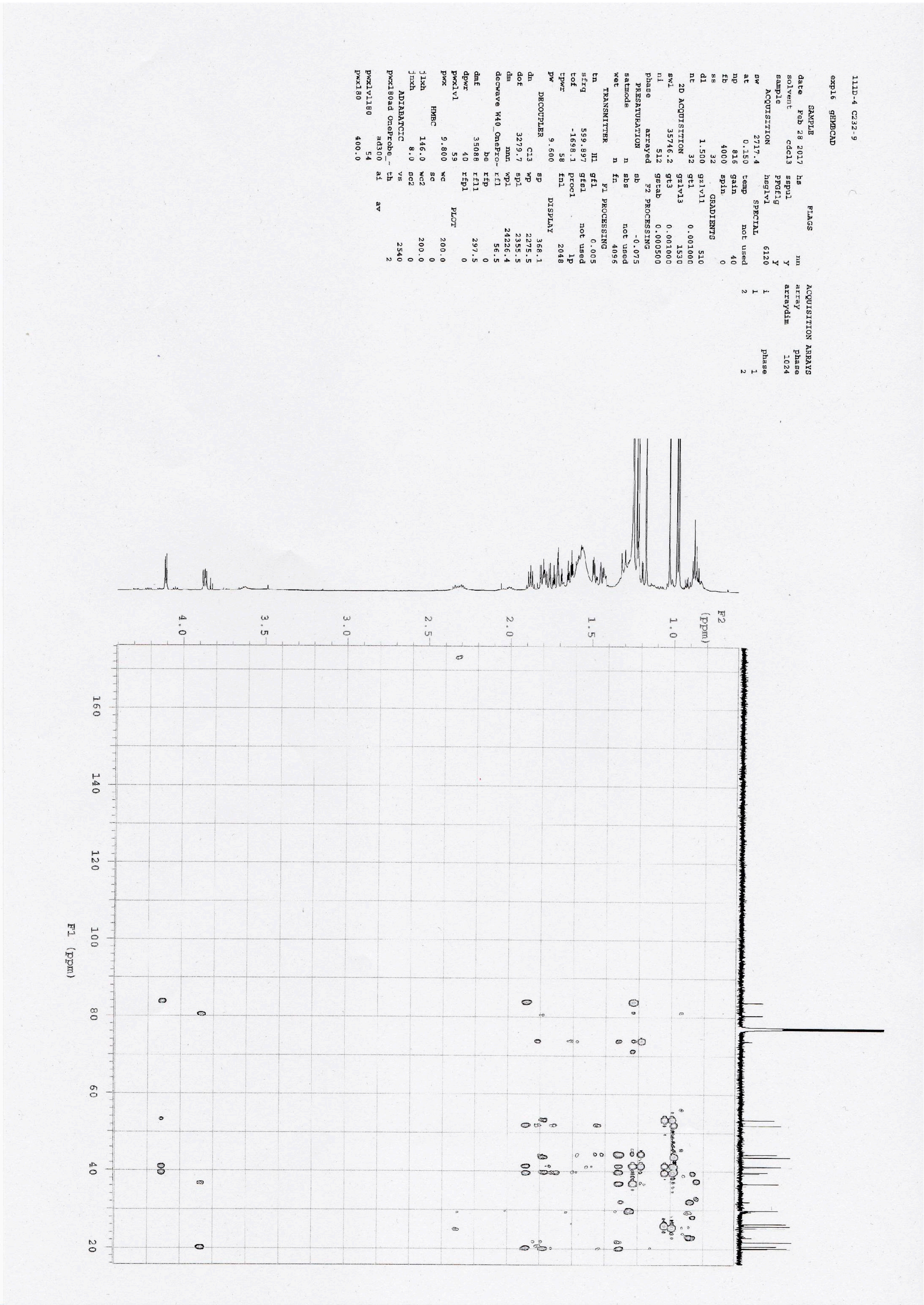

**Figure S18.** HMBC of **3**.

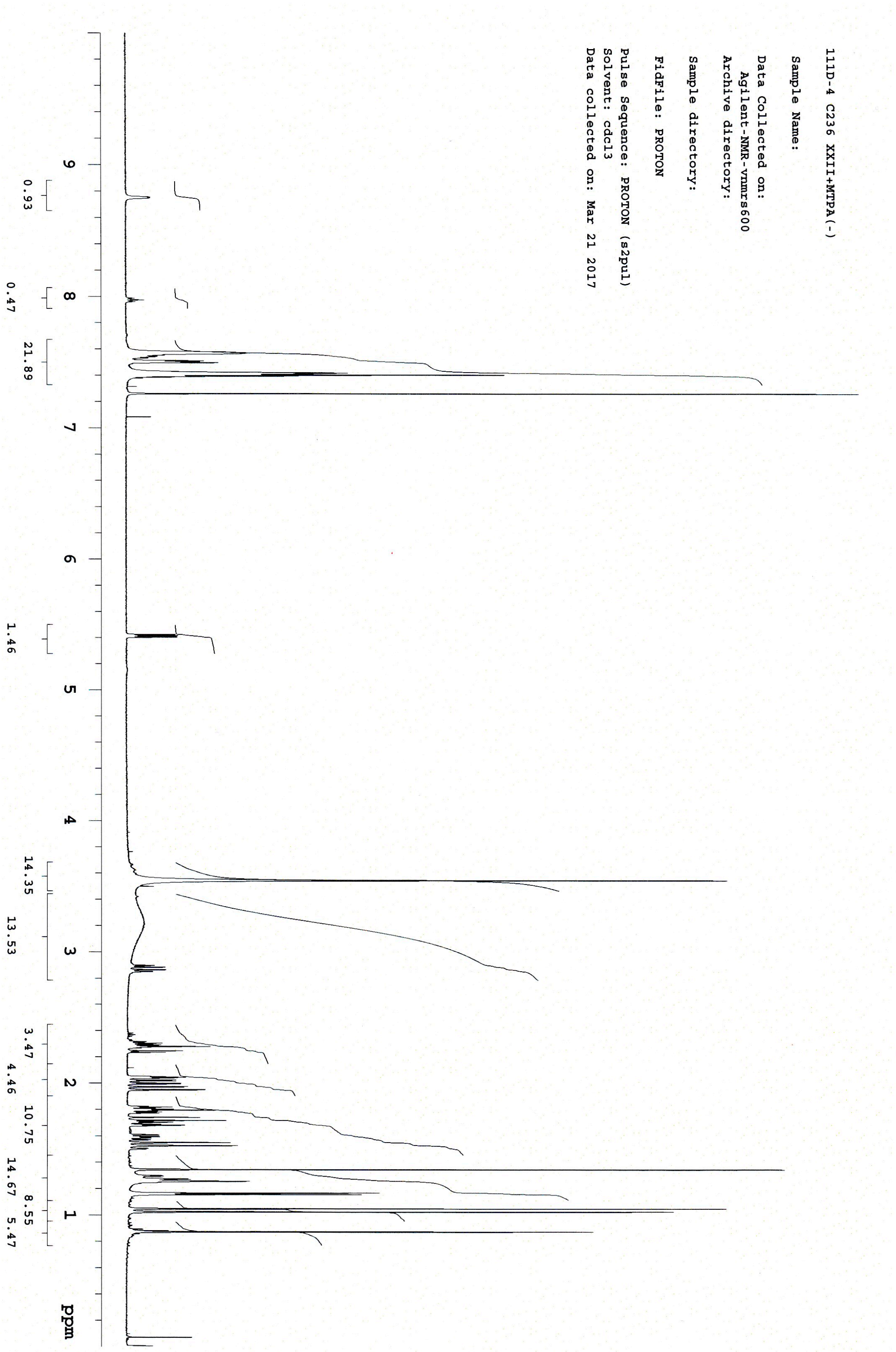


**Figure S19.** $^1$H NMR spectra of **1a** in $CDCl_3$.

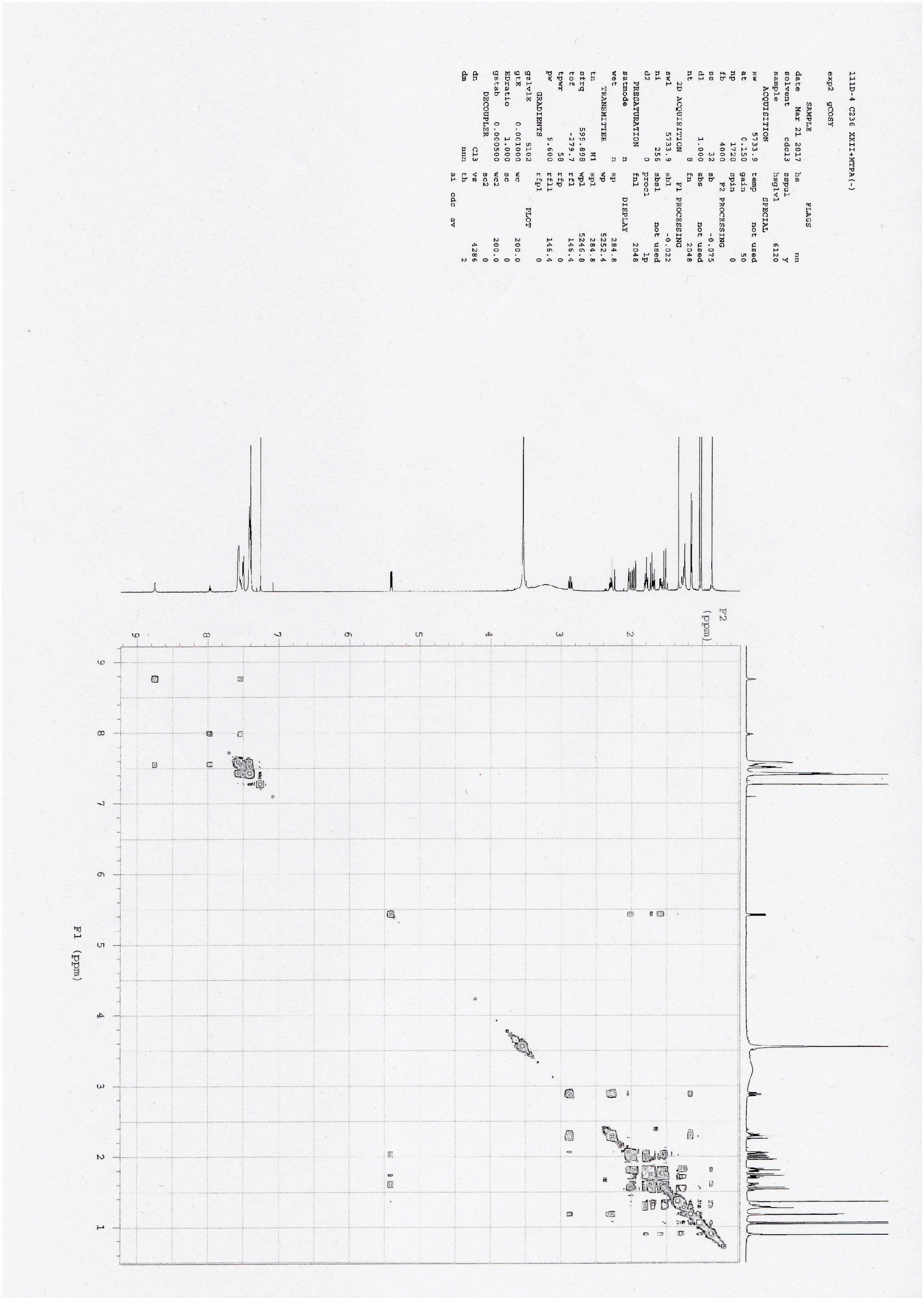

**Figure S20.** $^{1}H$-$^{1}H$ COSY of **1a**.

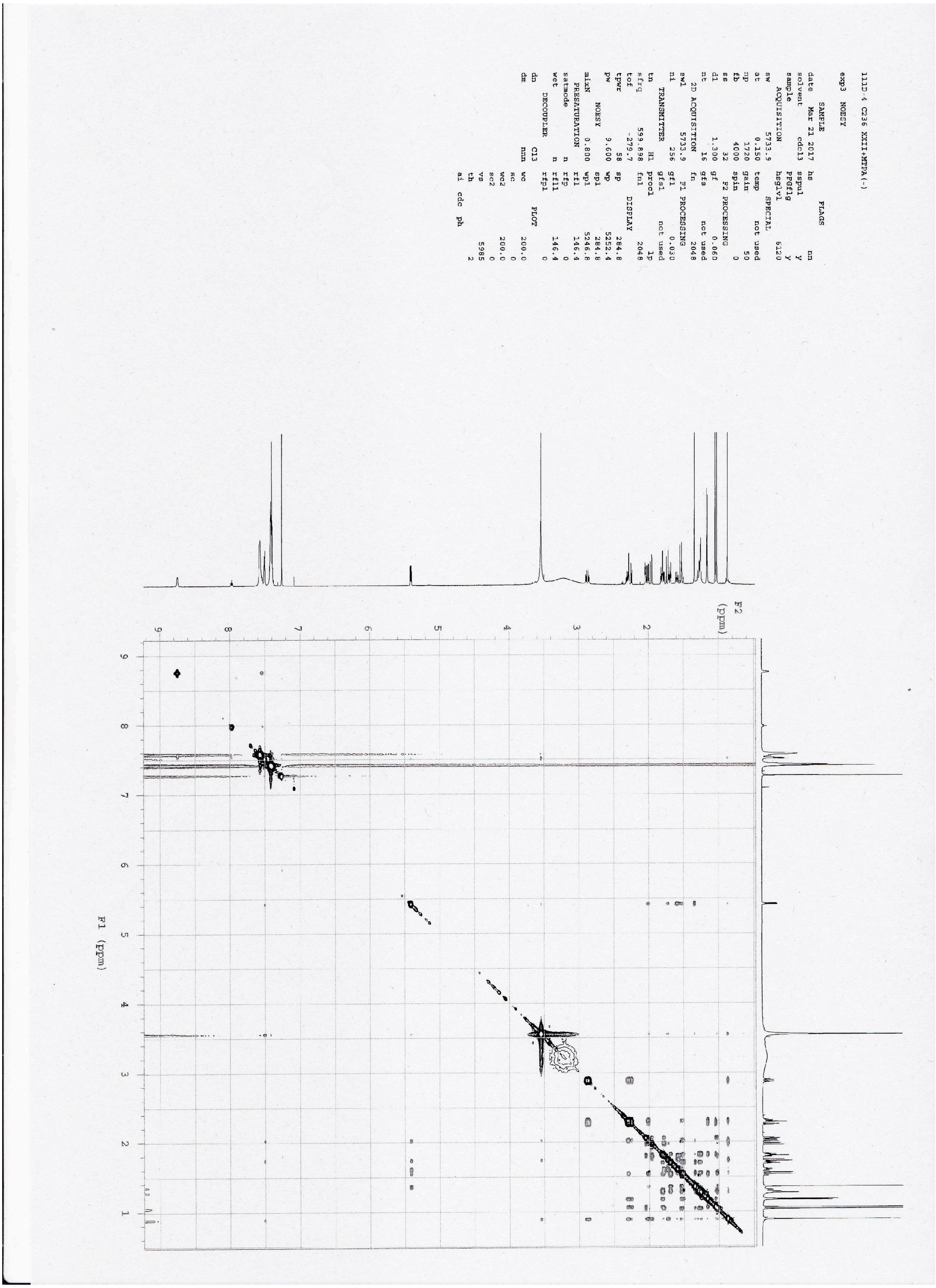

**Figure S21.** NOESY of **1a**.

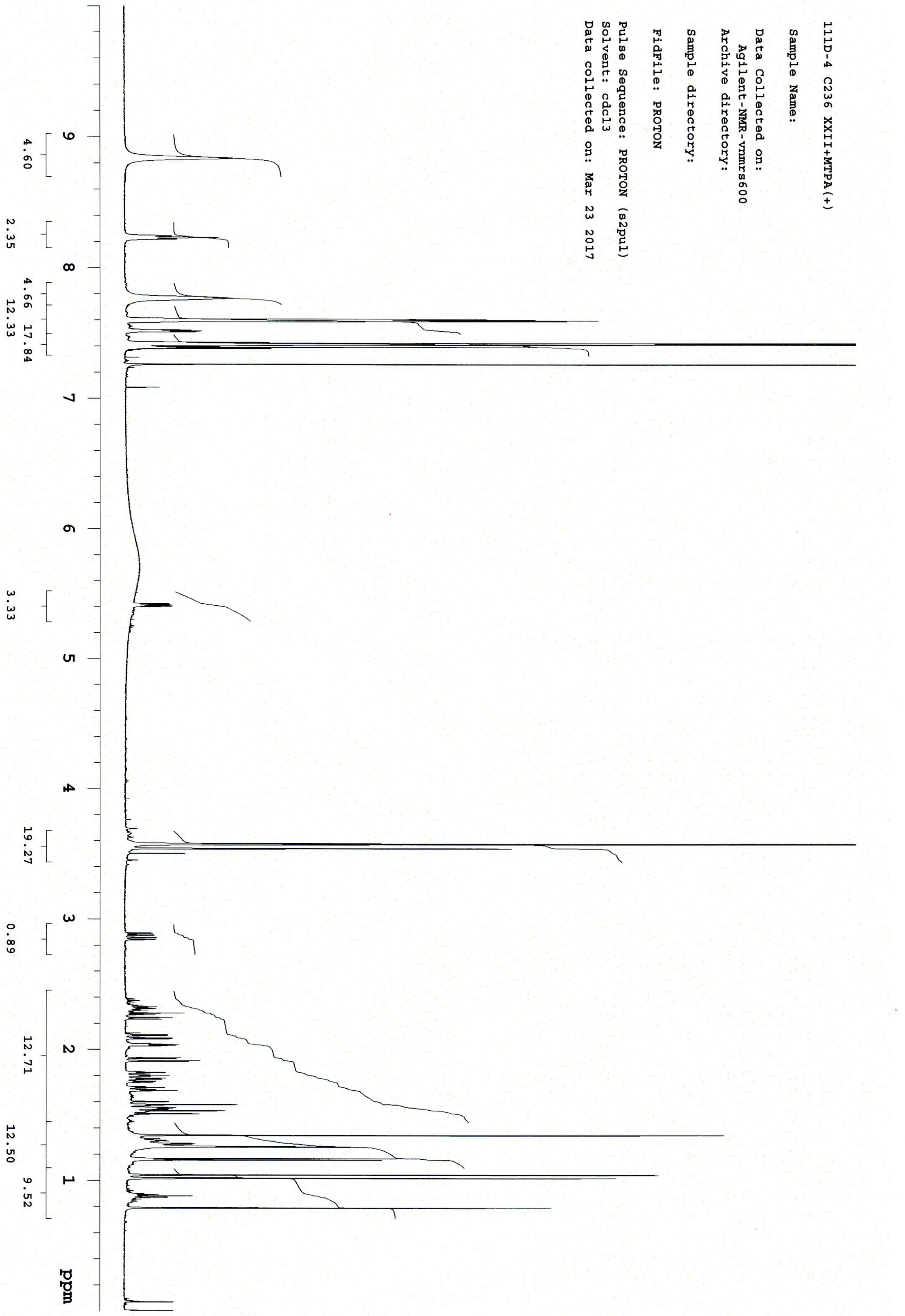


**Figure S22.** $^1$H NMR spectra of **1b** in $CDCl_3$.

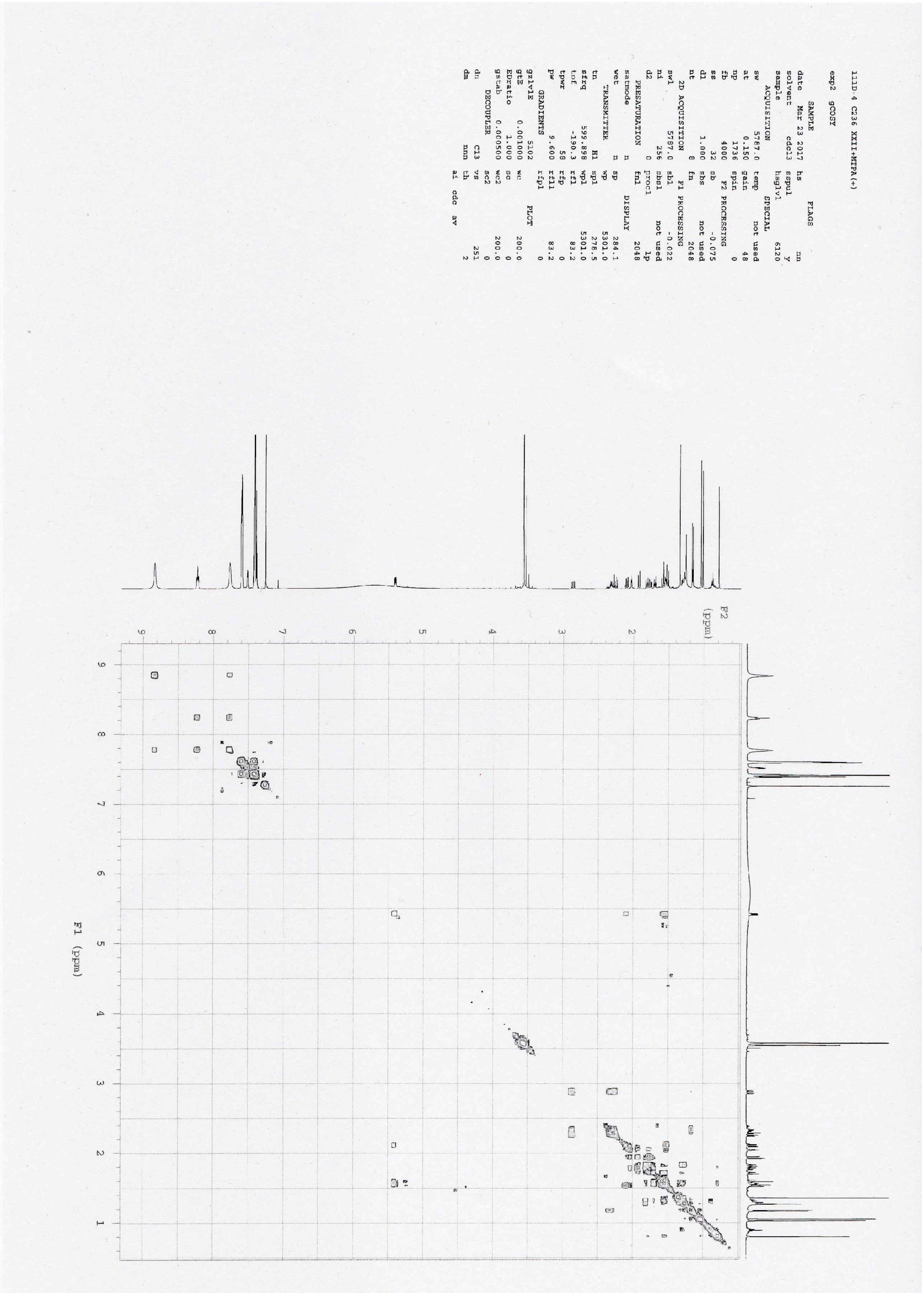

**Figure S23.** $^{1}$H-$^{1}$H COSY of **1b**.

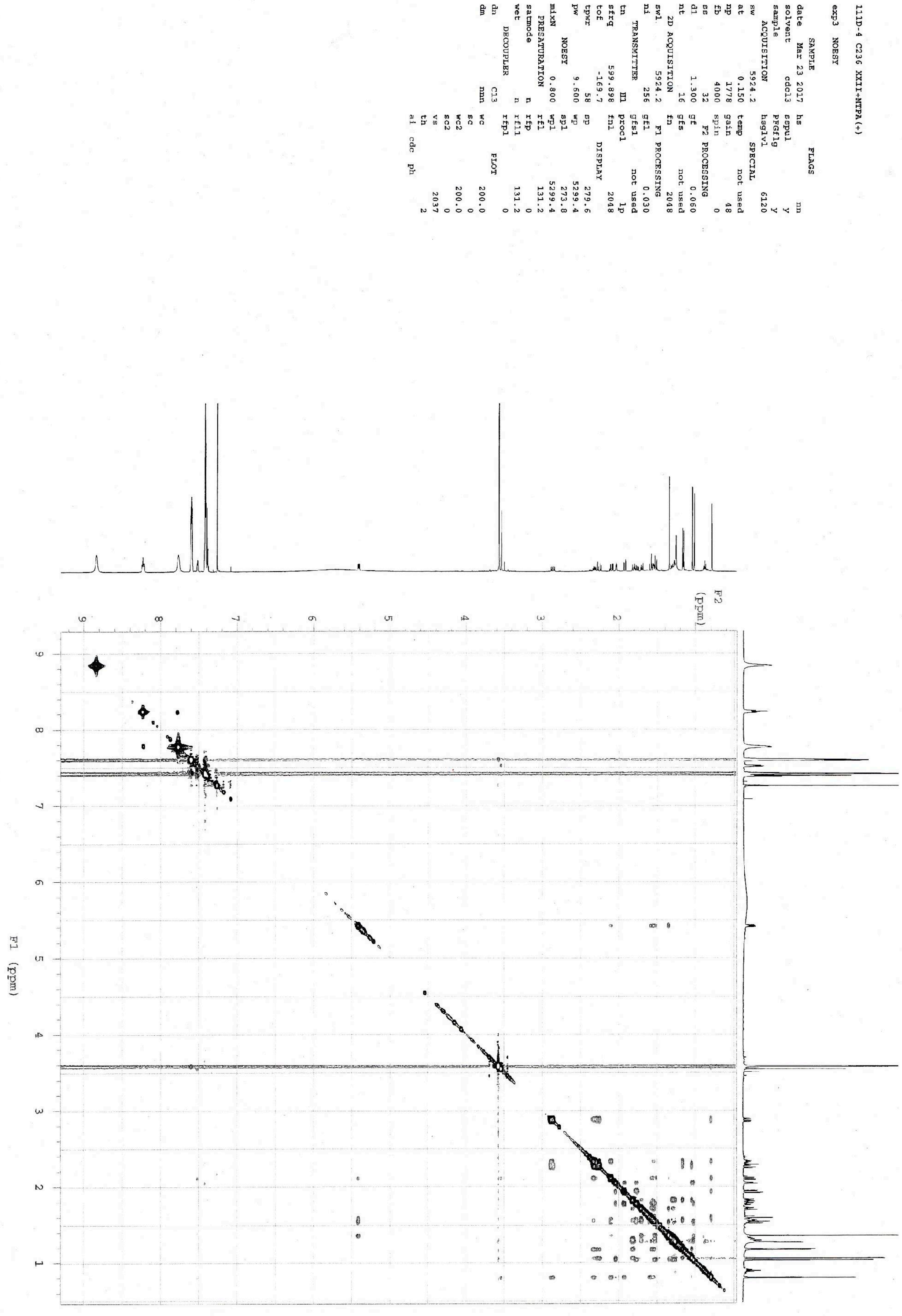


**Figure S24.** NOESY of **1b**.

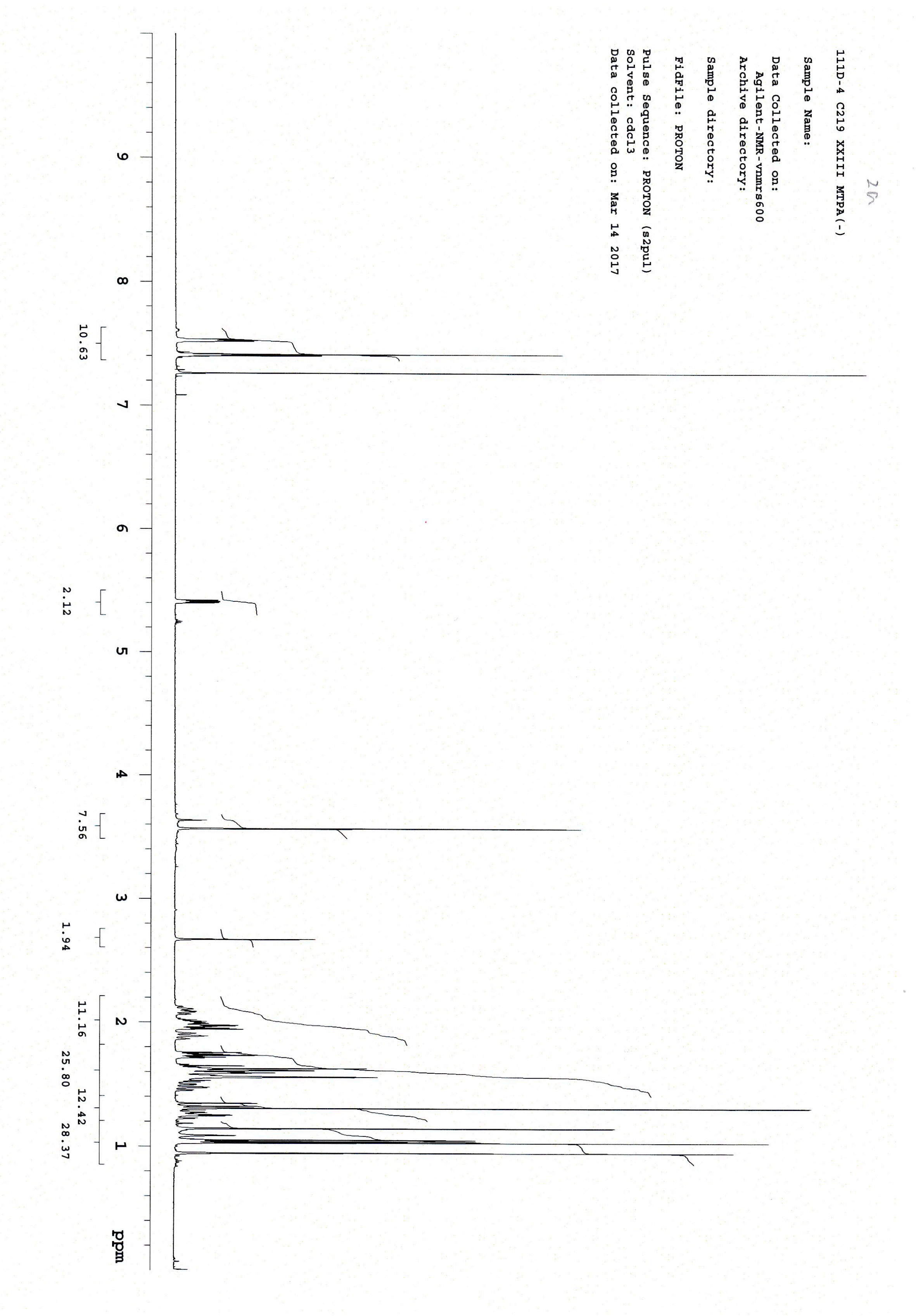


**Figure S25.** $^1$H NMR spectra of **2a** in $CDCl_3$.

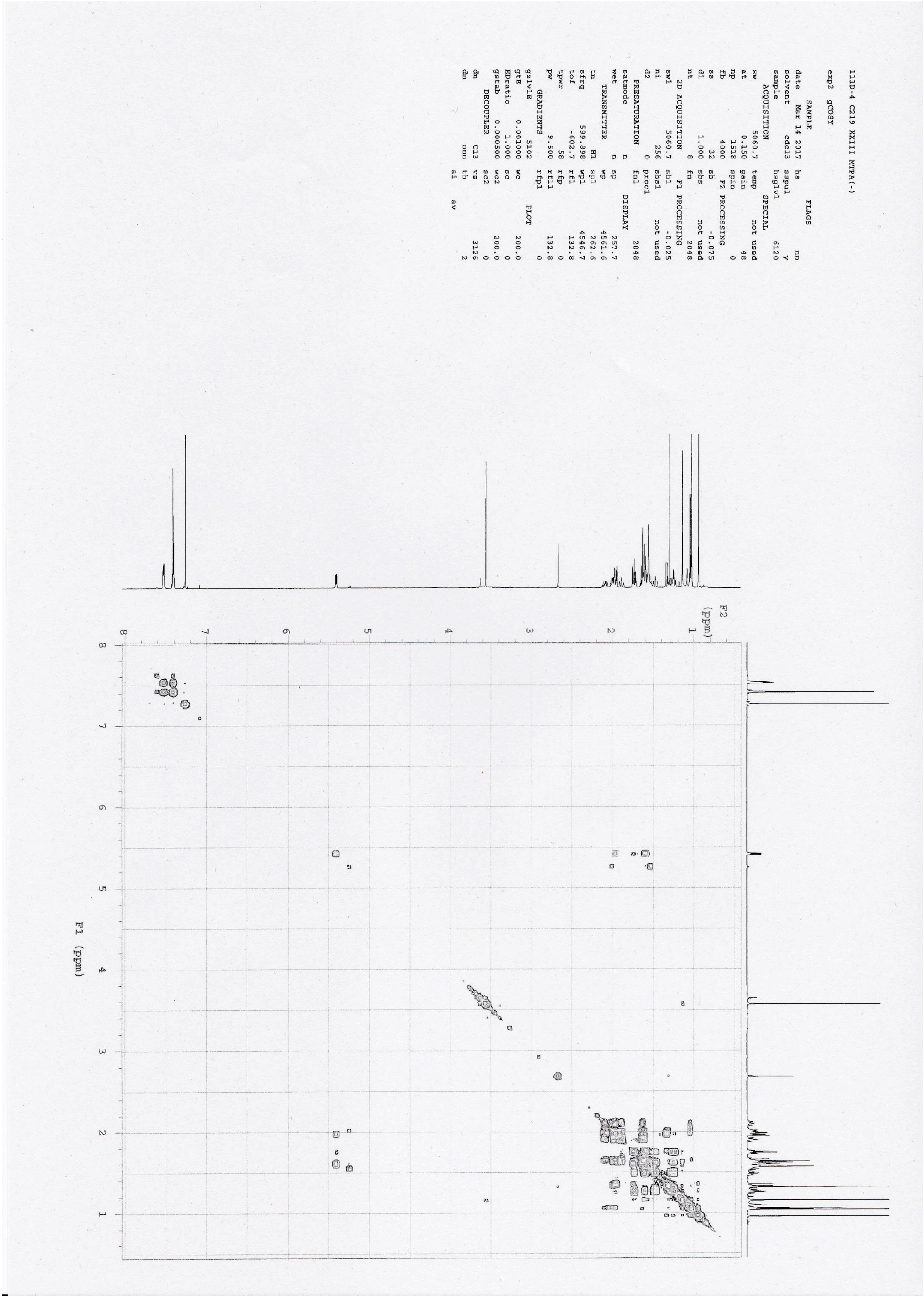


**Figure S26.** $^{1}$H-$^{1}$H COSY of **2a**.

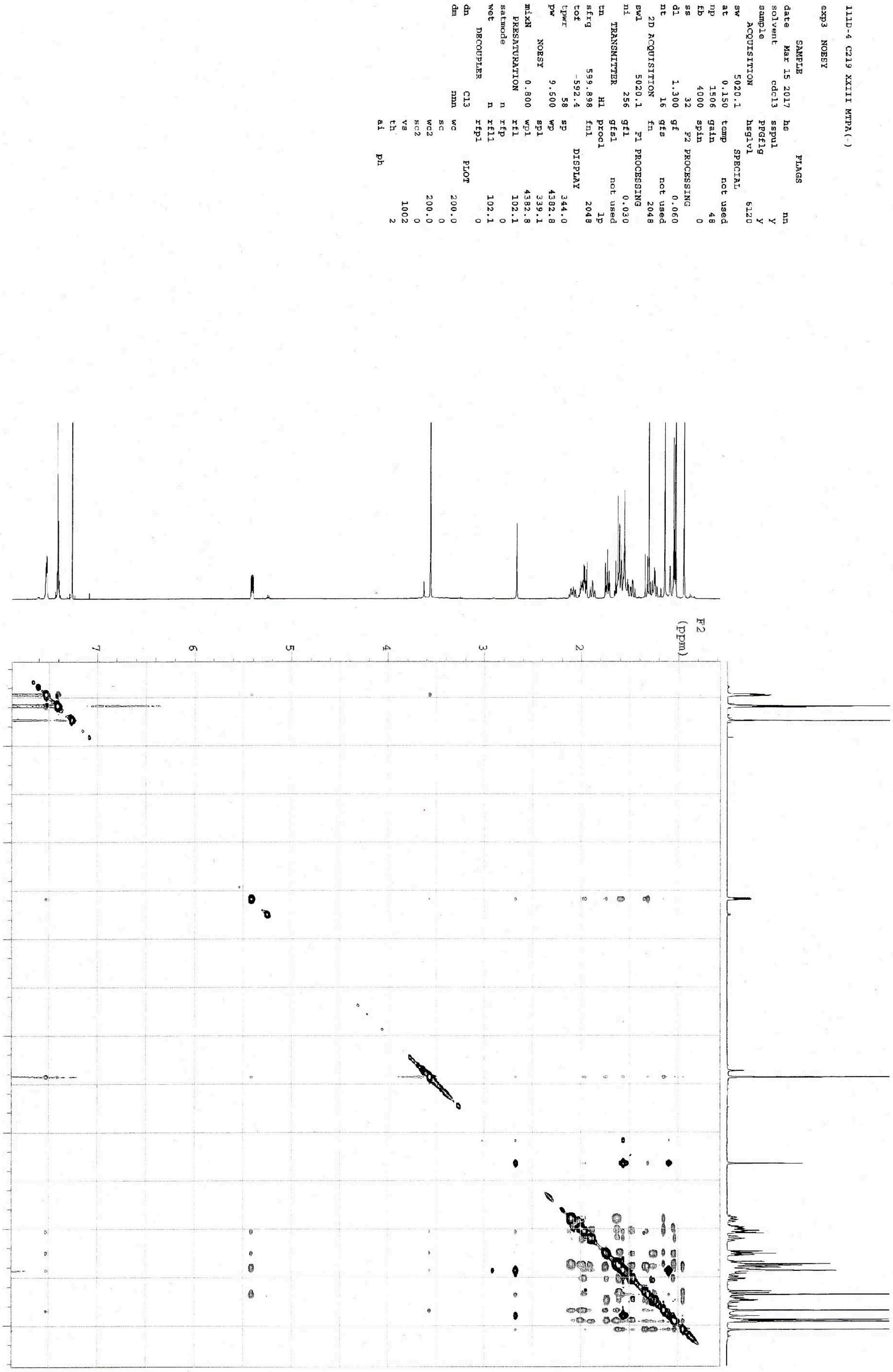


**Figure S27.** NOESY of **2a**.

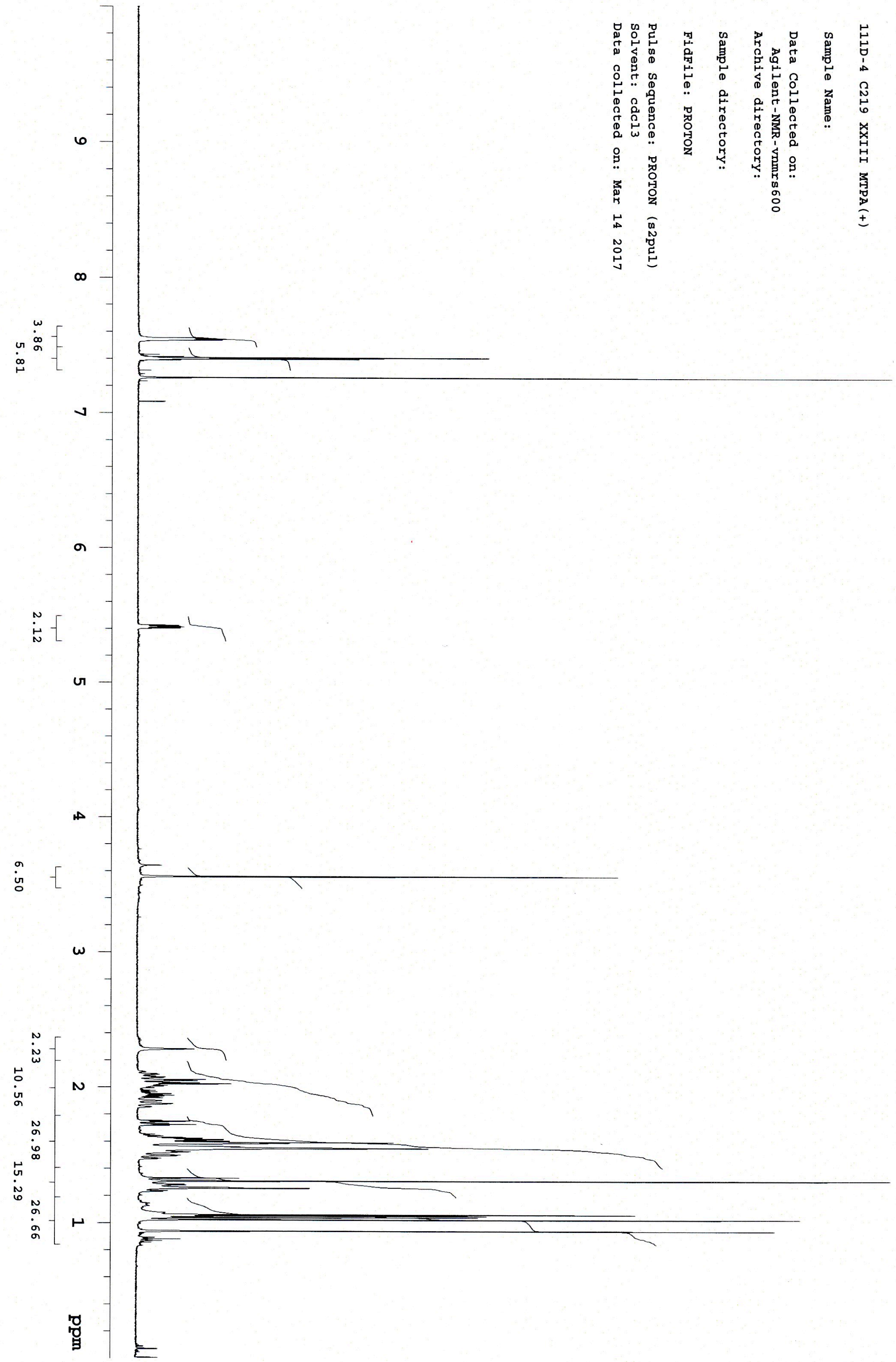


**Figure S28.** $^{1}H$ NMR spectra of **2b** in $CDCl_3$.

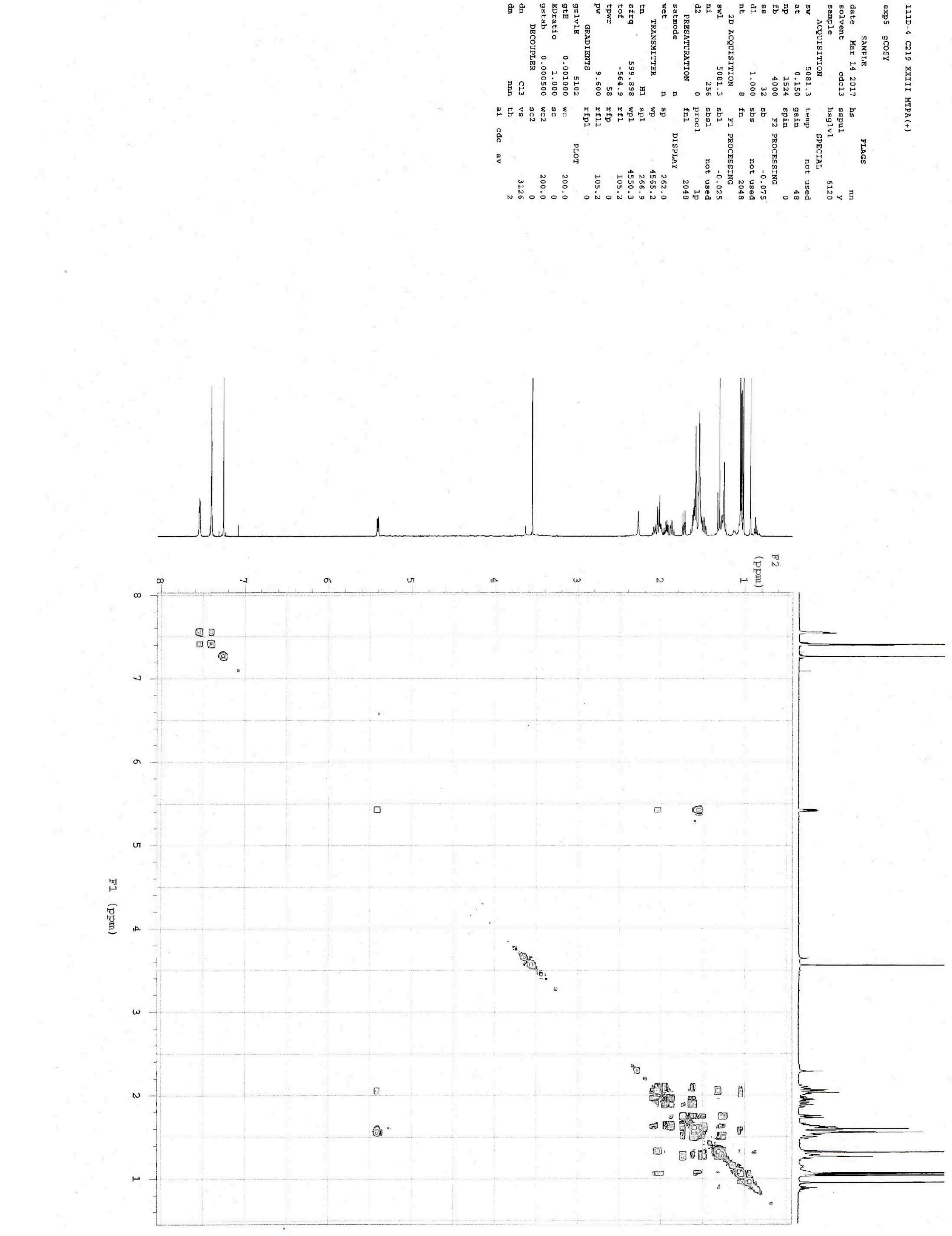


**Figure S29.** $^1$H-$^1$H COSY of **2b**.

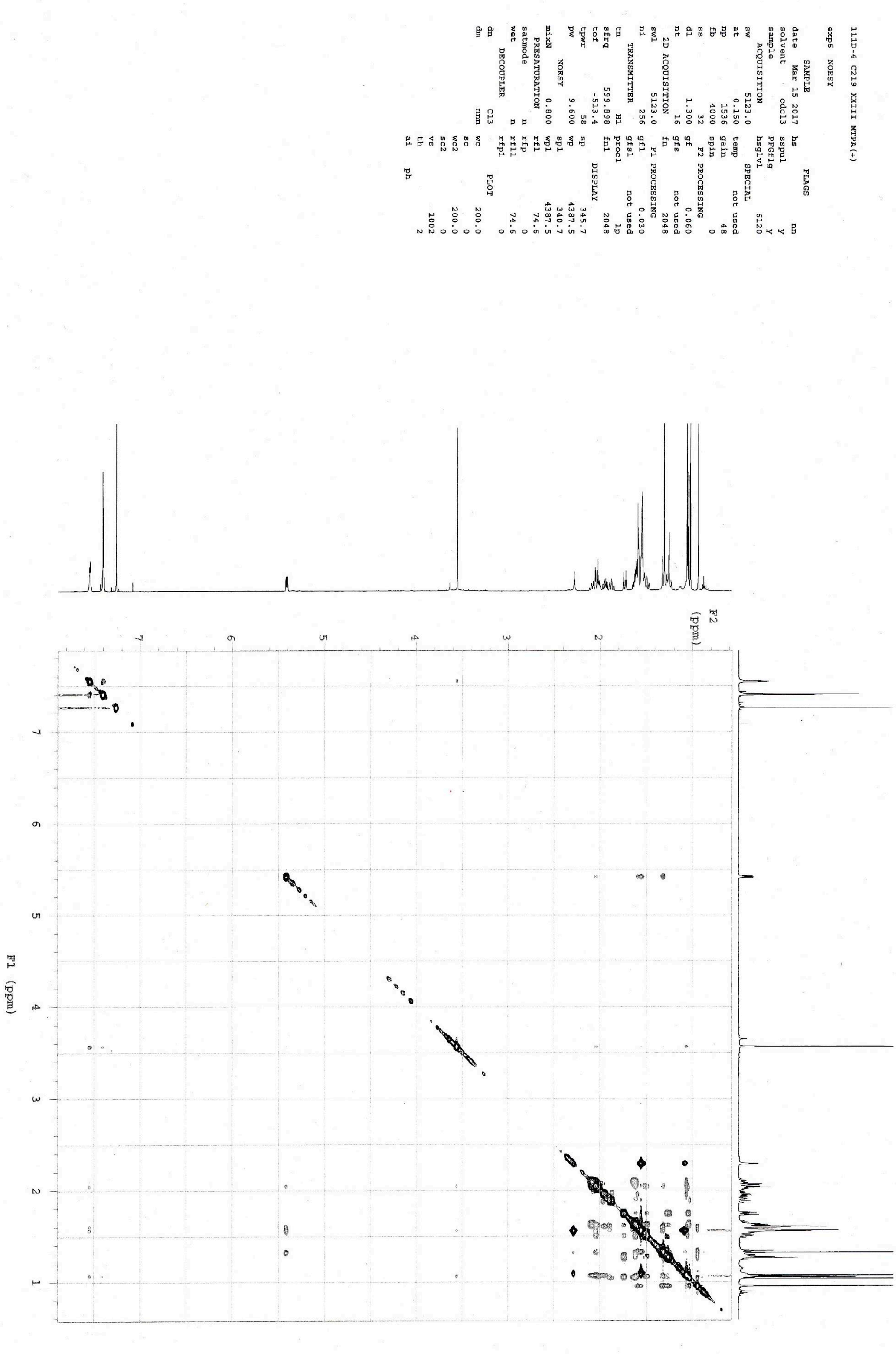


**Figure S30.** NOESY of **2b.**
